# β-adrenergic signaling triggers enteric glial reactivity and acute enteric gliosis during surgery

**DOI:** 10.1186/s12974-023-02937-0

**Published:** 2023-11-08

**Authors:** Patrick Leven, Reiner Schneider, Linda Schneider, Shilpashree Mallesh, Pieter Vanden Berghe, Philipp Sasse, Jörg C. Kalff, Sven Wehner

**Affiliations:** 1 Department of Surgery, University Hospital Bonn, Venusberg-Campus 1, 53127 Bonn, Germany; 2 Laboratory for Enteric NeuroScience (LENS), Translational Research Center for Gastrointestinal Disorders (TARGID), University of Leuven, Louvain, Belgium; 3 Institute of Physiology I, Medical Faculty, University of Bonn, Bonn, Germany

**Keywords:** Enteric glia, *RiboTag*, Gut inflammation, Postoperative ileus, Sympathetic nervous system, Neuroimmunology, Adrenergic Signaling

## Abstract

**Background:**

Enteric glia contribute to the pathophysiology of various intestinal immune-driven diseases, such as postoperative ileus (POI), a motility disorder and common complication after abdominal surgery. Enteric gliosis of the intestinal *muscularis externa* (*ME*) has been identified as part of POI development. However, the glia-restricted responses and activation mechanisms are poorly understood. The sympathetic nervous system becomes rapidly activated by abdominal surgery. It modulates intestinal immunity, innervates all intestinal layers, and directly interfaces with enteric glia. We hypothesized that sympathetic innervation controls enteric glia reactivity in response to surgical trauma.

**Methods:**

*Sox10*^*iCreERT2*^*/Rpl22*^*HA/*+^ mice were subjected to a mouse model of laparotomy or intestinal manipulation to induce POI. Histological, protein, and transcriptomic analyses were performed to analyze glia-specific responses. Interactions between the sympathetic nervous system and enteric glia were studied in mice chemically depleted of TH^+^ sympathetic neurons and glial-restricted *Sox10*^*iCreERT2*^*/JellyOP*^*fl/*+^*/Rpl22*^*HA/*+^ mice, allowing optogenetic stimulation of β-adrenergic downstream signaling and glial-specific transcriptome analyses. A laparotomy model was used to study the effect of sympathetic signaling on enteric glia in the absence of intestinal manipulation. Mechanistic studies included adrenergic receptor expression profiling in vivo and in vitro and adrenergic agonism treatments of primary enteric glial cell cultures to elucidate the role of sympathetic signaling in acute enteric gliosis and POI.

**Results:**

With ~ 4000 differentially expressed genes, the most substantial enteric glia response occurs early after intestinal manipulation. During POI, enteric glia switch into a reactive state and continuously shape their microenvironment by releasing inflammatory and migratory factors. Sympathetic denervation reduced the inflammatory response of enteric glia in the early postoperative phase. Optogenetic and pharmacological stimulation of β-adrenergic downstream signaling triggered enteric glial reactivity. Finally, distinct adrenergic agonists revealed β-1/2 adrenoceptors as the molecular targets of sympathetic–driven enteric glial reactivity.

**Conclusions:**

Enteric glia act as early responders during post-traumatic intestinal injury and inflammation. Intact sympathetic innervation and active β-adrenergic receptor signaling in enteric glia is a trigger of the immediate glial postoperative inflammatory response. With immune-activating cues originating from the sympathetic nervous system as early as the initial surgical incision, adrenergic signaling in enteric glia presents a promising target for preventing POI development.

**Supplementary Information:**

The online version contains supplementary material available at 10.1186/s12974-023-02937-0.

## Background

The enteric nervous system (ENS), consisting of enteric neurons and enteric glia, is a branch of the autonomous nervous system that governs various functions throughout the alimentary tract, such as gastric motility, fluid homeostasis, and blood flow [1]. Enteric glia are diverse neuroglia, displaying several subtypes based on morphology and location in intestinal structures [1]. Most enteric glia also show a unique co-expression pattern of SRY-Box transcription factor 10 (SOX10) together with either the glial markers S100B or glial fibrillary acidic protein (GFAP) [2], or proteolipid protein 1 (PLP1) [2, 3].

At first, enteric glia were mainly considered solely as neuron-supporting cell populations of the ENS, providing nutrition and protection for enteric neurons [1]. More recent studies provided new insights into enteric glia involvement in gastrointestinal (GI) homeostasis [1] and discovered their vital role in chronic [4] and acute [5] gut inflammation. Enteric glia switch to a reactive state during gut inflammation, altering their morphology, expression pattern, and functional character [1]. So far, broader enteric glial reactivity in POI has only been analyzed in vivo in the full tissue context, also described as a POI-related “enteric gliosis”, but cell-intrinsic molecular responses of enteric glia and their primary activating mechanism were still missing. We termed the reactive inflammatory tissue state of the muscularis externa tissue “enteric gliosis” [5] as it shares molecular expression patterns with tissue gliosis in the central nervous system (CNS). Notably, this CNS gliosis is defined by the reactivity of glial cells, such as microglia, oligodendrocytes, and most importantly, astrocytes [6], the counterpart to enteric glia, which become activated during neuroinflammation in chronic disease states [7, 8] and after neurological traumata [9].

Part of the enteric gliosis state are reactive enteric glia, which modulate their microenvironment by secreting cytokines and chemokines like interleukin (IL)-6, C–C motif chemokine ligand (CCL)-2 [5, 10], and C–X–C motif chemokine ligand (CXCL)-10 [11], pro-inflammatory molecules, such as nitric oxide [12], and molecules that elicit an anti-inflammatory response, including glial cell-derived neurotrophic factor (GDNF) [13] and S-Nitrosoglutathione [14].

Although a comprehensive picture of reactive enteric glia in intestinal inflammation is still missing, recent studies provide evidence about stimuli being able to induce gliosis in the gut. These include lipopolysaccharide (LPS) [15], cytokines such as interleukin (IL)-1β [10], tumor necrosis factor (TNF)α [16], and interferon-gamma (IFNγ) [17], as well as purines, e.g., ADP [4] or ATP [5]. The latter is released by both intrinsic and extrinsic enteric neurons innervating the gut and is co-released with norepinephrine (NE) from sympathetic neurons. NE is the principal neurotransmitter of the sympathetic nervous system (SNS) [18], known to interact with enteric glia through adrenergic receptors [19]. More recently, several publications highlighted the involvement of the SNS in inflammation-based infectious [20] and non-infectious bowel diseases [21] and its ambivalent effect on the inflammatory milieu, depending on the disease stage, neurotransmitter concentration, and receptor binding [22].

We recently showed that the SNS affects the postoperative inflammatory immune cell milieu in postoperative ileus (POI) and functionally impacts the disease progression [23]. POI is a frequent transient GI-motility disorder and complication of abdominal surgery. Patients with POI suffer from nausea, abdominal distension pain, reduced oral food tolerance, delayed recovery, and finally, an extended hospitalization phase with a high medico-economic burden on our health care systems [24]. A prominent response to abdominal surgery is a dysbalance of the sympathetic and parasympathetic inputs of the intestine towards sympathetic overactivation. Notably, this sympathetic overactivity is already induced by the skin incision [25], and the subsequent surgical manipulation of the intestine (or other visceral organs) enhances this overactivity. Hallmarks of enteric gliosis have been identified as part of POI (5, 10, 26) and are discussed to be of potential value for therapeutic interventions in POI and other intestinal inflammation-driven diseases [26]. As SNS-released mediators directly act on enteric glia [19, 27], we hypothesized that adrenergic signaling might be an early trigger of acute postoperative enteric glial reactivity in the onset phase of POI.

To test this hypothesis, we used *Sox10*^*iCreERT2*^*/Rpl22*^*HA/*+^ mice to extract cell-specific mRNA from hemagglutinin-labeled ribosomes of enteric glia [28, 29] within three phases of POI: the immediate initiation phase, the manifestation, and the resolution phase. We found striking evidence of strong enteric glial reactivity in the immediate postoperative phase. Furthermore, we discovered that laparotomy, the first step of abdominal surgery, which does not include manipulation of any other visceral organs, is sufficient to trigger enteric glial activation in the intestine. Sympathetic denervation studies, live calcium imaging in ex vivo ganglia, enteric glia-restricted optogenetic activation of β-adrenergic downstream signaling in *Sox10*^*iCreERT2*^*/JellyOP*^*fl/*+^*/Rpl22*^*HA/*+^ mice, and stimulation of primary EGC cultures gave insight into the distinct β-adrenergic signaling pathways triggering enteric glial reactivity in POI.

## Materials and methods

### Materials

#### Animals

*Sox10*^*iCreERT2*^ (*B6-Tg(Sox10-icre/ERT2)388Wdr/J*) mice were crossbred with *Rpl22*^*HA/*+^ (*B6N.129-Rpl22tm1.1Psam/J*)/ tdTomato (*B6;129S6-Gt(ROSA)26Sortm14(CAG-tdTomato)Hze*) mice. Additionally, *Sox10*^*iCreERT2*^*/RiboTag/tdTomato* were crossbred with *JellyOP* mice (*CD1-Gt(ROSA)26Sor*^*em1(CAG−JellyOp−eGFP)*^; Additional file 1: Method S2, S3) [30] for optogenetic activation experiments. Animals were housed under SPF conditions in the central housing facility or our laboratory (Immunpathophysiology, University Hospital Bonn, Bonn, Germany). Male mice (10–12 weeks) were used in the intestinal manipulation and laparotomy experiments, and mice of both sexes (10–20 weeks) were used for optogenetic activation experiments and pharmacological modification with reserpine and tyramine carried out under German federal law (Az.: 81-02.04.2016 A367 and 81-02-04-02018.A221, 84–02.04.2017.A114).

Inducible Cre was activated by intraperitoneal injections of 100 µl Tamoxifen [MP Biomedicals, Irvine, CA, USA] dissolved in 10% ethanol and 90% sterile corn oil (final concentration 10 mg/ml) for three consecutive days. Experiments were performed one week after the last injection.

Calcium imaging studies were conducted on female *Wnt1-Cre; R26R-GCaMP3* mice as approved by the Animal Ethics Committee of the University of Leuven (Belgium) in the laboratory of Pieter Vanden Berghe.

#### In vivo optogenetic activation of adrenergic signaling in enteric glia

*Sox10*^*iCreERT2*^*/RiboTag/tdTomato*/*JellyOP* animals and *JellyOP*-negative littermate controls received pain medication (Tramadol [Grünenthal, Aachen, NRW, DE]; i.p.) 15 min before surgery. During surgery, animals were anesthetized with Isoflurane and kept on a heating pad to stabilize body temperature. After abdominal shaving, the abdominal cavity was opened (2 cm incision) along the *linea alba* and held open by clamps while the small bowel was gently lifted and placed on gauze. The *JellyOP* construct was activated with supramaximal blue light (470 nm, > 0.5 mW/mm^2^) at a distance of 10 cm for 15 min with regular moisturization with saline. Activation of the *JellyOP* construct triggers a G_s_ signaling cascade downstream from 1D4, that resembles activation by β-adrenergic stimulation. The intestine was gently replaced, and the opened cavity was sutured. Animals were replaced in their cages and slowly woke from the narcosis under heating lamps during the following 30 min. Animals received further pain medication orally (Tramadol [Aliud Pharma, Laichingen, BW, DE]) via their water supply.

#### Post-operative ileus (POI) model

**Fig. 1:**
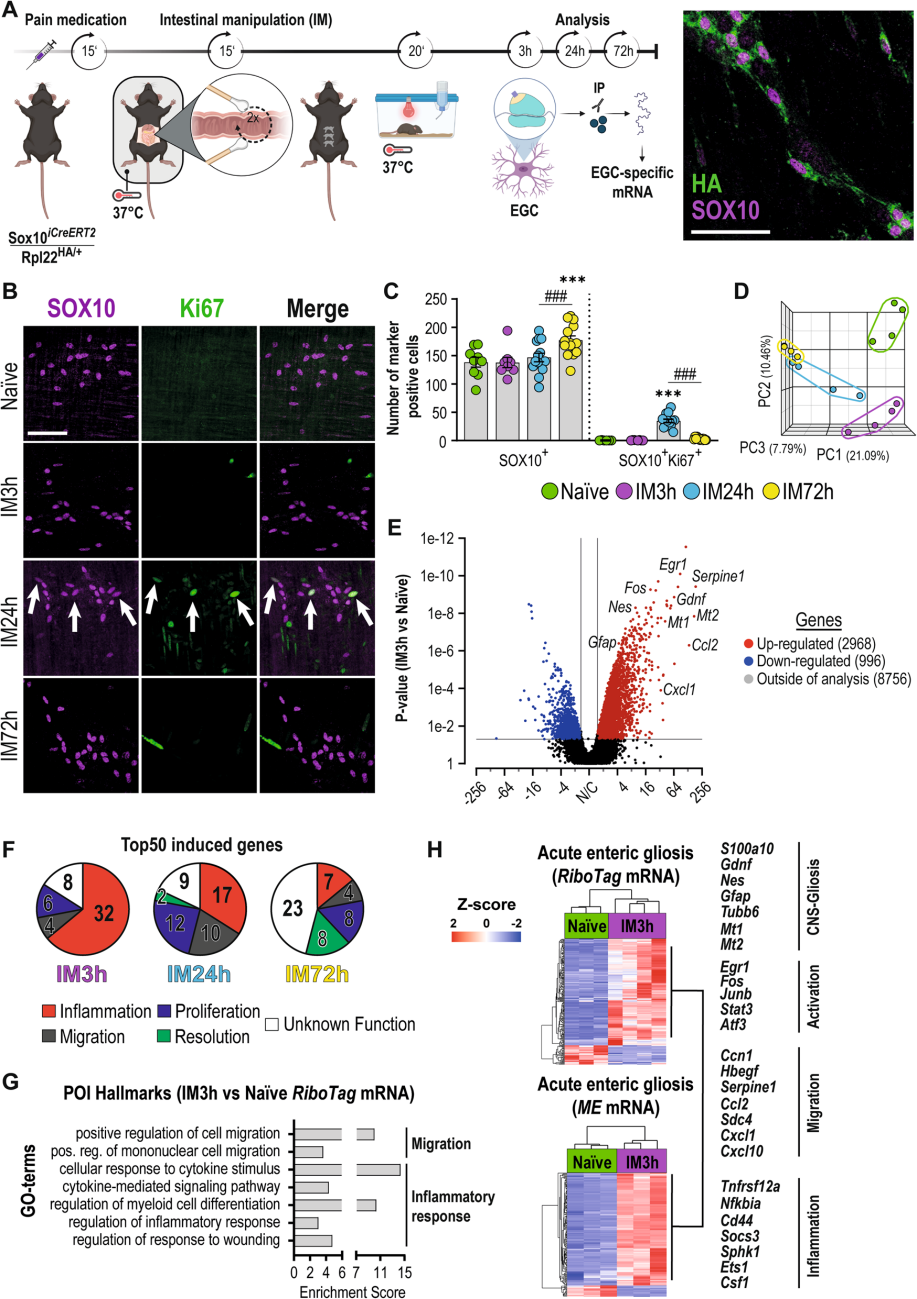
Enteric glia react to mechanical stimuli and transition into an acute gliosis state. **A** Schematic description of the surgical procedure (intestinal manipulation, IM) with follow-up *RiboTag* approach in *Sox10*^*iCreERT2*^*/Rpl22*^*HA/*+^ enteric glia and immunohistological image of HA (green) and SOX10 (magenta) co-expression in *ME*. Scale bar (100 µm). The *RiboTag* procedure was performed 3 h, 24 h, or 72 h after surgery. **B** Confocal images of SOX10 (magenta) and Ki67 (green) expression in whole mounts of small bowel *ME* at different time points. Scale bar (100 µm). **C** Histological analysis (mean ± SEM) of SOX10^+^ and SOX10^+ ^Ki67^+^ cells per field of view (n = 6–14 animals per time point; mean counts of 5 images per n ± SEM; two-way ANOVA, to naive *** < 0.01, between IM24/IM72h ### < 0.01). **D** Principal component analysis (PCA) of a bulk RNA-Seq of *Sox10*^*iCreERT2*^*/Rpl22*^*HA/*+^ *RiboTag* mRNA at different time points. **E** Volcano plot for actively transcribed genes at IM3h in *Sox10*^*iCreERT2*^*/Rpl22*^*HA/*+^enteric glia with significantly differentially transcribed genes (p-value < 0.05, >  ± twofold) marked in red (upregulated) and blue (downregulated) and annotation of notable genes. **F** Venn diagrams of the top 50 induced genes at IM3h, IM24h, and IM72h separated into clusters. **G** Analysis of enriched GO-terms in mRNA from *Sox10*^*iCreERT2*^*/Rpl22*^*HA/*+^enteric glia for POI hallmarks related to migration and inflammatory response. **H** RNA-Seq heat maps for naïve and IM3h samples of *Sox10*^*iCreERT2*^*/Rpl22*^*HA/*+^*RiboTag* mRNA and total RNA “acute enteric gliosis” induction and an indication of genes related to key POI hallmarks. (n = 3–4 animals per time point)

Animals received pain medication (Tramadol [Grünenthal, Aachen, NRW, DE]; i.p.) 15 min before surgery. During surgery, animals were anesthetized with Isoflurane and kept on a heating pad to stabilize body temperature. After abdominal shaving, the abdominal cavity was opened (2 cm incision) along the *linea alba* and held open by clamps, while the small bowel was gently lifted and placed on gauze (Fig. 1A). The small bowel was mechanically manipulated by light pressure with moist cotton swaps in a rolling motion towards the *Caecum* (2x). The intestine was gently replaced, and the opened cavity was sutured. Additionally, we performed a modified laparotomy model in which no manipulation was performed. Animals were replaced in their cages and slowly woke from the narcosis under a red light during the following 30 min. Animals received further pain medication orally (Tramadol [Aliud Pharma, Laichingen, BW, DE]) via their water supply.

#### Gastrointestinal transit

Animals received 100 µl of FITC-dextran [Sigma Aldrich, St. Louis, MO, USA] via gavage and rested for 90 min without additional food or water. Subsequently, animals were sacrificed, intestines eventrated, and separated into segments (stomach 1; small bowel 2–11, ~ 3 cm each; caecum 12; colon 13–15, ~ 2 cm each). Segments were flushed with Krebs–Henseleit buffer (Additional file 1: Table S1), and eluates were analyzed for FITC fluorescence. The geometric center was calculated to generate GI-transit time for naïve, Lap 24 h, IM 24 h, and IM 72 h animals.

#### Sympathetic denervation

*Sox10*^*iCreERT2*^*/Rpl22*^*HA/*+^*mice* were injected with 250 µl 6-Hydroxydopamine (6-OHDA; 80 mg/kg body weight in saline [Sigma Aldrich, St. Louis, MO, USA]) for three consecutive days as described before [23]. Animals rested for fourteen days after the final injection before subsequent experiments were performed.

#### Pharmaceutical adrenergic modulation (reserpine and tyramine)

*Sox10*^*iCreERT2*^*/Rpl22*^*HA/*+^mice were injected s.c. with 100 µl reserpine (20 mg/kg body weight in saline [#83,580, Sigma Aldrich, St. Louis, MO, USA]) adapted from [31] and kept for 24 h. Animals subsequently underwent laparotomy as described above (with and without prior administration of reserpine) and were sacrificed 3 h later.

*Sox10*^*iCreERT2*^*/Rpl22*^*HA/*+^ mice were injected i.p. with 100 µl tyramine (100 mg/kg body weight in saline [#W421501, Sigma Aldrich, St. Louis, MO, USA]) adapted from [32] or 100 µl saline and sacrificed 3 h later.

#### Primary murine enteric glial cell (EGC) cultures

Primary EGC cultures were generated from small bowel *muscularis externa (ME)* of 8–12-week-old *Sox10*^*iCreERT2*^*/Rpl22*^*HA/*+^*/Ai14*^*fl/fl*^ mice. Briefly, the intestine was eventrated, flushed with oxygenated Krebs–Henseleit buffer (cell culture; Additional file 1: Table S1), dissected into 3–5 cm long segments, and transferred to ice-cold, oxygenated Krebs–Henseleit buffer. *ME* tissue was mechanically separated from the mucosal layer, centrifuged (300 g, 5 min), and digested in dissociation buffer (Additional file 1: Table S1) in a water bath (15 min, 37 °C, 150 rpm). The enzymatic reaction was stopped by the addition of 5 ml DMEM + 10% FBS [Sigma Aldrich, St. Louis, MO, USA], centrifugation (300 g, 5 min), and resuspension in proliferation media (Additional file 1: Table S1). Cells were kept in proliferation media for 7 days (37 °C, 5% CO_2_) before dissociation with trypsin (0.25%, 5 min, 37 °C) [Thermo Fisher Scientific, Waltham, MA, USA] and seeding on Poly-L-Ornithine [Sigma Aldrich, St. Louis, MO, USA] coated 6-well plates at 50% confluence in differentiation media (Additional file 1: Table S1). Cells were differentiated for seven days before treatment with norepinephrine (NE; 10 µM, 100 µM), adrenergic receptor (AR) agonists (β-AR/isoprenaline (1 µM, 10 µM); α2a-AR/guanfacine (10 µM); β3-AR/CL-316243 (10 µM) [all Tocris Bioscience, Bristol, UK]), or forskolin [#HY-15371, MedChemExpress, Monmouth Junction, NJ, USA] in PBS for 3 h or 24 h. Cell culture constituted mainly of enteric glia (> 85%) and small amounts of fibroblasts (< 10%), described in more detail in [5]. Conditioned media was used for ELISA analysis, and cells were processed for RNA analysis.

#### In vitro optogenetic activation of adrenergic signaling in enteric glial cell cultures

*JellyOP*^fl/+^ animals were used to isolate primary enteric glial cells as described above. Cells were transfected with 1 µl (1.69 × 10^8^ VG/ml) of an rAAV2/1-hGFAP-NLS-Cre virus construct (“AAV-GFAP-Cre”; Additional file 1: Method S4) to activate the JellyOP construct and subsequently differentiated for seven days. Differentiated cells were subjected to four consecutive 1 min pulses of blue light (470 nm, 32 µW/mm^2^) in a custom-built illuminator for cell culture plates. Media from illuminated and dark-kept cells was used for ELISA.

#### Calcium imaging

Female *Wnt1-Cre;R26R-GCaMP3* mice were killed by cervical dislocation, as approved by the Animal Ethics Committee of the University of Leuven (Belgium). These mice express the fluorescent Ca^2+^ indicator GCaMP3 in all enteric neurons and glia [33, 34].

The ileum was carefully removed, opened along the mesenteric border, and pinned flat in a sylgard-lined dissection dish in cold O_2_/CO_2_ (95%/5%) suffused Krebs buffer (120.9 mM NaCl; 5.9 mM KCl; 1.2 mM MgCl_2_; 1.2 mM NaH_2_PO_4_; 14.4 mM NaHCO_3_; mM 11.5 Glucose; 2.5 mM CaCl_2_). The mucosa, submucosa, and circular muscle were removed by microdissection to expose the myenteric plexus. These preparations were stabilized over an inox ring using a matched o-ring [35], which was mounted in a cover glass bottom chamber on the microscope stage. 3D recordings of the GCaMP3 were made on an inverted spinning disk confocal microscope (Nikon Ti—Andor Revolution—Yokogawa CSU-X1 Spinning Disk [Andor, Belfast, UK]) with a Nikon 20 × lens (NA 0.8), excitation 488 nm and detection 525/50 nm. A Piezo Z Stage controller (PI) was used to record fast 3D stacks at 2 Hz. Tissues were constantly supplied with oxygenated Krebs buffer via a gravity-fed perfusion system that allowed instantaneous switching between control and high K^+^, Substance P (10^–5^ M, to identify the glia network, [36]) or isoprenaline (10^–5^ M) containing Krebs buffer. The tissues were constantly perfused by O_2_/CO_2_ (95%/5%) suffused Krebs buffer containing 2 µM nifedipine to prevent most of the muscle contractions.

#### Analysis of calcium imaging

All calcium image analysis was performed with custom-written routines (available via [37]) in Igor Pro [Wavemetrics, Lake Oswego, OR, USA]. Image registration was performed in Fiji using the descriptor-based registration algorithm developed by Preibish et al. [38]. Registered images were further analyzed in Igor 8, and regions of interest were drawn to extract temporal information. Peak amplitude calculation was performed using custom-written procedures as previously described [37, 39]. The average Ca^2+^ signal intensity was calculated, normalized to the initial GCaMP3 signal, and reported as F/F_0_.

#### Immunohistochemistry

Immunohistochemistry was performed on terminal ileum parts. Briefly, the ileum was placed in Sylgard gel-covered Petri dishes and opened along the mesentery. After fixation with 4% PFA for 20 min, mucosal-free *ME* whole mounts were prepared through mechanical separation of both layers. Next, whole mounts were permeabilized (1% Triton X-100/PBS; RT, 20 min) and blocked with (5% donkey serum, 0.25% Triton X-100/PBS; RT, 1 h) before antibody incubation (primary: 4 °C, overnight; secondary: 1.5 h, RT; Additional file 1: Table S2).

#### Microscopy imaging

Widefield images used for quantitative analysis of proliferation, numbers of SOX10 positive cells, and MPO infiltration were obtained on a Nikon Eclipse TE2000-E with a magnification of 20 × and a field of view of 397 µm × 317 µm or a Nikon Eclipse T*i*2 with a magnification of 20 × and a field of view of 769 µm × 769 µm. Representative images are confocal slices obtained with a Leica SP8 AOTF confocal microscope using a 40 × objective.

#### Hanker-Yates histology

Hanker-Yates staining was performed on the terminal ileum. Briefly, the ileum was pinned to Sylgard gel-covered Petri dishes and opened along the mesentery. After fixation with pure ethanol for 10 min, mucosal-free *ME* whole mounts were prepared by mechanically separating both layers. Before mounting, whole mounts were subjected to Hanker-Yates myeloperoxidase staining solution (RT, 10 min).

#### Western Blot

A BCA protein assay kit [Thermo Fisher Scientific, Waltham, MA, USA] was used to assess protein lysate concentrations. SDS-PAGE was performed with 100 µg of protein. The primary and secondary antibodies (Additional file 1: Table S2) were incubated overnight at 4 °C and 1 h at RT, respectively.

#### ELISA

Conditioned media from EGC cultures treated with NE or agonists for the indicated time points was collected, centrifuged (5000*g*, 5 min), and snap-frozen in liquid nitrogen. According to the manufacturer's instruction manual, media was analyzed for IL-6 release with an ELISA kit [R&D Systems, Abingdon, GB].

#### RiboTag approach

*RiboTag* immunoprecipitation was performed according to a previously established protocol (Additional file 1: Method S1, [28]). Briefly, the muscle layer of the whole small bowel tissue was mechanically separated from the mucosal layer and placed in RNA*later* [Thermo Fisher Scientific, Waltham, MA, USA]. Muscle tissue was lysed on a Precellys homogenizer [Bertin Instruments, Montigny-le-Bretonneux, FR] (3 × 5000 rpm, 45 s; 5 min intermediate incubation on ice) in pre-cooled homogenization buffer (Additional file 1: Table S1), centrifuged (10 min, 10,000*g*, 4 °C), and supernatants saved. “Input” controls were generated from 50 µl cleared lysate. Samples were incubated with anti-HA antibody (5 µl; 1 mg/ml; Additional file 1: Table S2; 4 h, 4 °C, 7 rpm). Lysate/Antibody conjugates were added to 200 µl of washed A/G dynabeads [Thermo Fisher Scientific, Waltham, MA, USA] and incubated (overnight, 4 °C, 7 rpm). Beads were washed thrice with high salt buffer (Additional file 1: Table S1). Ribosomes containing specific mRNA were eluted from beads, and RNA was extracted with a Qiagen micro kit.

#### cDNA Synthesis and quantitative PCR Analysis

Purified RNA (10 µg) was transcribed with the Applied Biosystems™ High-Capacity cDNA Reverse Transcription Kit [Applied Biosystems, Foster City, CA, USA] according to the manufacturer's instruction manual. cDNA (1:10 diluted) was added to SYBR™ Green PCR Master Mix [Applied Biosystems, Foster City, CA, USA] and analyzed by qPCR [Applied Biosystems, Foster City, CA, USA] (Additional file 1: Table S3).

#### RNA-Seq analysis

Libraries were prepared with QuantSeq 3′ mRNA-Seq Library Prep Kit [Lexogen, Greenland, NH, USA] and sequenced (single-end 50 bp, 10 M reads) on an Illumina Hiseq 2500. “Partek Flow” software was used to analyze RNA-Seq data (Lexogen pipeline 12,112,017), and Ensemble transcripts release 99 for mm10 mouse alignment. The pipeline consisted of two adapter trimming and a base-trimming step with subsequent quality controls (QC). Reads were aligned with star2.5.3, followed by a post-alignment QC, and quantification to an annotation model. Normalized counts were subjected to principal component and gene set analysis. Pipeline information can be found within our uploaded sequencing files (*GSE198889*).

#### Statistical analysis

Statistical analysis was performed with Prism 9.0 [GraphPad, San Diego, CA, USA] using Student's t-test, multiple unpaired t-test, one-way, or two-way ANOVA as indicated in the figure legends. Significance to controls is resembled by *, while significance to other samples is indicated by ^#^. All plots are mean ± SEM. Animals for experiments were age- and sex-matched and randomly assigned to the experimental groups.

## Results

### Intestinal inflammation induces enteric gliosis during post-operative ileus development

In a previous study, we re-defined the inflammatory state of the post-operative *ME*, containing reactive enteric glia, based on a publication-based gene selection associated with the term “gliosis” in the CNS [5] and termed this condition “enteric gliosis”. As the underlying gene selection only defined the overall transcriptional inflammatory responses of the enteric glial-containing tissue but did not reflect individual cell-type-specific changes of enteric glia, we aimed to precisely determine their reactivity and better understand their role during acute inflammation. Therefore, we assessed the transcriptional changes of enteric glia by a *RiboTag* approach. The *RiboTag,* in conjunction with a *Sox10*^*iCreERT2*^ system (Fig. 1A, [28]), enabled the isolation of actively transcribed mRNA from enteric glia-specific HA-tagged ribosomes and subsequent RNA-Seq analysis with glial mRNA (Additional file 2: Fig. S1A). Enteric glia reactivity was induced through a standardized model of intestinal inflammation resulting in post-operative ileus (POI). A proper tissue response was confirmed by our previously defined enteric gliosis gene panel [5]. POI progresses in three stages, and the selection of representative time points 3 h, 24 h, and 72 h after intestinal manipulation (IM) enabled us to define the molecular responses within the early/immediate phase (IM3h), inflammatory/manifestation phase (IM24h), and recovery/resolution phase (IM72h). IM (Fig. 1A) triggered acute gut inflammation in the *muscularis externa* (*ME*) (Additional file 2: Fig. S1B), with a decrease in gastrointestinal motility (Additional file 2: Fig. S1C) and an induction of a substantial influx of infiltrating leukocytes (Additional file 2: Fig. S1D) peaking at IM24h, confirming that *Sox10*^*iCreERT2*^* RiboTag* mice develop POI. The existence of enteric gliosis was shown by increased protein expression of GFAP and vimentin (VIM) during POI progression (Additional file 2: Fig. S1E). Notably, the number of Sox10^+^Ki67^+^ enteric glia in the *ME* increased at IM24h (35 ± 3 vs. 0.17 ± 0.15 cells/ field of view) but not at IM3h (0.5 ± 0.18 cells/ field of view). SOX10^+^ Ki67^+^ enteric glia numbers dropped to baseline levels at IM72h (3 ± 0.6 cells/ field of view) (Fig. 1B, C), indicating the presence of a timely-limited trigger of glial cell proliferation in the acute phase of inflammation. Supportively, total numbers of SOX10^+^ enteric glia increased at IM72h compared to naïve and IM24h time points (Fig. 1C), showing that molecular features of proliferation indeed resulted in increased EGC numbers in the recovery phase. These changes coincided with POI hallmarks and an overall strong transcriptional response related to inflammation in the *ME* (Additional file 2: Fig. S1B).

Principal component analysis of *RiboTag* samples revealed a clear separation of gene expression patterns at investigated POI time points (Fig. 1D). Volcano plots comparing naïve with IM samples showed the most substantial enteric glial activation at IM3h with 2968 genes up- and 996 genes down-regulated (Fig. 1E), compared to IM24h (101 up- and 1703 down-regulated genes) and IM72h (42 up- and 2218 down-regulated genes) (Additional file 2: Fig. S1F, G). Validation of the top 50 induced genes at all disease time points with gene databanks (*GeneCards* and *Mouse Genome Informatics*) revealed a change of enteric glia toward an inflammation-related cell type (Fig. 1F). Enteric glia displayed a strong expression of inflammatory genes (32 of the top 50; e.g., metallothioneins *Mt1* and *Mt2*, *Tnfrsf12a*, *Nfkbia*, *Sphk1*) in the initial phase that declined during POI progression and was replaced by a strong expression of migratory (10 of the top 50; e.g., *Ccl2*, *Ccl6*, *Ccl9*) and proliferation-associated genes (12 of the top 50; e.g., *Mcm3*, *Chaf1a*) at IM24h. A so far undefined resolution phenotype arose at IM72h (8 resolution genes of the top 50), showing the induction of olfactory receptors (e.g., *Olfr373*, *Olfr95*, *Olfr1254*), implicated in gut inflammation [40] (Fig. 1F). Notably, the number of actively transcribed genes pulled down with the *RiboTag* approach was also the highest during disease onset at IM3h (12,720 genes) compared to IM24h (8129 genes) and IM72h (3599 genes) (Additional file 2: Fig. S1H). The high transcriptional activity at IM3h aligns with the early enteric glial reactivity. GO analyses showed the enrichment of genes associated with multiple immunological aspects and POI hallmarks, including migration regulation, cytokine signaling, and myeloid cell differentiation (Fig. 1G). To define inflammation-induced enteric glial activation on a transcriptional level, we generated the novel gene ontology (GO) term "acute enteric gliosis" (Additional file 1: Table S4). Therein, we validated the expression of published gliosis genes, previously defined by our group [5], in the *RiboTag* data set and added highly induced genes at IM3h (> tenfold vs. naïve; e.g., *Serpine1, Mt2, Gdnf*) together with genes that were only detected in samples at the IM3h time point (naïve 0 counts; IM3h > 5 counts per sample; e.g., *Fosl, Ucn2, Areg*). Notably, around half of the published gliosis genes are also expressed by enteric glia during POI (Additional file 1: Fig. S1I, Additional file 2: Table S4). Application of this novel GO term showed strong induction of gliosis genes in enteric glia 3 h after manipulation with a steep decline at IM24h and IM72h (Additional file 2: Fig. S1J). To test the applicability of the "acute enteric gliosis" GO term as an indicator of acute enteric gliosis in the full *ME* tissue, we analyzed an RNA-Seq data set generated of total *ME* from POI mice. The resulting heat map mirrored the prominent induction of gliosis genes from our *RiboTag* analysis at IM3h (Additional file 2: Fig. S1K), with most of the upregulated gliosis genes induced exclusively in the early disease phase (IM3h, 94 genes), some overlapping genes at IM3h and IM24h (49 genes; Additional file 2: Fig. S1L) and only one induced gene overlapping between IM3h and IM72h. Highly induced genes at IM3h included known astrogliosis genes (*Gfap*, *Nes, Mt1, Mt2, Gdnf*), early response genes (*Egr1*, *Fos*), migration factors (*Ccl2*, *Cxcl1, Cxcl10, Serpine1*), and inflammatory factors (*Nfkbia, Socs3, Sphk1, Cd44*) (Fig. 1H). Notably, almost no acute enteric gliosis gene panel genes were upregulated in the *ME* at later postoperative time points, supporting its usefulness as an acute enteric gliosis marker panel.

These data provide evidence of the strong plasticity of enteric glia during acute intestinal inflammation, wherein an acute immune-reactive phenotype is a very early event. At the disease peak, 24 h after surgery, enteric glial reactivity switched towards a phenotype supporting migration and proliferation, which further declined towards a resolution type at IM72h.

### Sympathetic signaling triggers acute enteric glia gliosis

**Fig. 2:**
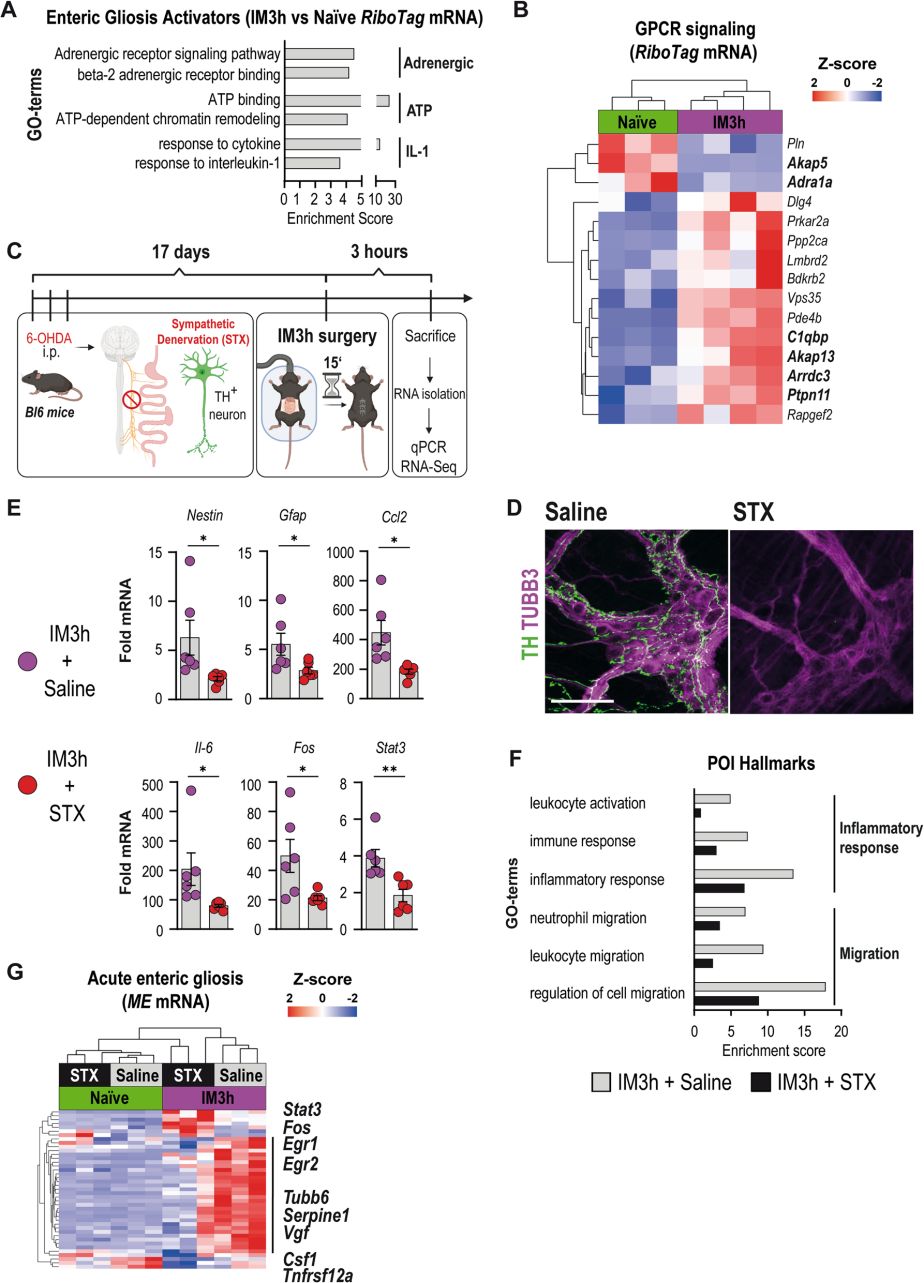
Acute enteric gliosis is modulated by sympathetic innervation. **A** Analysis of enriched GO-terms in mRNA from *Sox10*^*iCreERT2*^*/Rpl22*^*HA/*+^enteric glia 3 h after IM for POI hallmarks related to gliosis triggering pathways. **B** Heat map for the GO-term "GPCR signaling" in naïve and IM3h samples of mRNA from *Sox10*^*iCreERT2*^*/Rpl22*^*HA/*+^ enteric glia and with selected genes highlighted. **C** Schematic description of chemical sympathetic denervation of C57BL6 mice with three consecutive intraperitoneal 6-OHDA injections (days 1–3). After 14 days, mice underwent IM. *ME* was isolated three hours later (IM3h) for qPCR and RNA sequencing. **D** Confocal images of immunohistological stainings of TUBB3 (magenta) and TH (green) expression in whole mounts of control (saline) and 6-OHDA treated (sympathectomized/STX) small bowel *ME* 17 days after injection. (n = 6 animals per condition). Scale bar (100 µm). **E** qPCR analysis showing fold changes of mRNA levels (mean ± SEM) from IM3h/Saline and IM3h/STX mice for enteric gliosis-related genes (2^−ΔΔCT^, *18S*, IM3h + saline; n = 6 animals per condition; Student’s t-test, * < 0.05, ** < 0.01). **F** Analysis of enriched POI hallmark GO-terms in IM3h/Saline total RNA and comparatively reduced in IM3h/STX samples related to inflammatory response and migration. **G** RNA-Seq heat map of our “acute enteric gliosis” GO-term for naïve and IM3h samples treated with saline or 6-OHDA (STX), and an indication of STX-affected genes (black line)

The rapid transition of enteric glia into an inflammatory phenotype raised the question of which mechanisms might trigger this immediate enteric glial activation. A previous study from our group showed that extracellular ATP levels quickly rose after abdominal surgery, thereby triggering an enteric glial immune activation during POI [5]. As ATP is co-stored with norepinephrine (NE) in synaptic vesicles of sympathetic nerves and intestinal sympathetic activity is known to become immediately over-activated during surgery, we hypothesized that sympathetic pathways might contribute to the acute enteric gliosis phenotype. We performed a comparative GO-term analysis and found enriched expression of genes associated with “beta-2-adrenergic receptor binding” and “adrenergic signaling pathway” (Fig. 2A). Interestingly, the enrichment scores were comparable with GO terms linked to IL-1 signaling and ATP-guided expression changes, albeit drastically lower than general ATP binding, both pathways known to activate enteric glia upon surgery [5, 41]. Heat maps of differentially expressed genes related to G-protein-coupled receptor (GPCR) signaling showed a clear pattern between naïve and IM3h enteric glia in our *RiboTag* mice (Fig. 2B). Activation of enhanced adrenergic signaling was confirmed in *ME* tissue samples, depicting a similar gene expression pattern for adrenergic signaling activity (Additional file 2: Fig. S2A).

Since acute gliosis triggered changes in genes related to adrenergic signaling, we theorized that ablation of the intestinal sympathetic innervation affects acute enteric gliosis. To test this hypothesis, we chemically ablated sympathetic neurons (sympathectomy/STX). Denervation was facilitated by the established model of i.p. injection of 6-Hydroxydopamine (6-OHDA), starting 17 days before surgery (Fig. 2C), resulting in a complete depletion of TH^+^ neurons within the *ME* of C57BL6 wildtype mice (Fig. 2D). A proof-of-concept analysis by qPCR of *ME* RNA samples from IM3h animals showed a significant reduction of gliosis markers (*Nestin, Gfap*), early response genes (*Stat3, Fosb*), and pro-inflammatory mediators (*Il-6, Ccl2*) in denervated animals (Fig. 2E). Next, we re-analyzed an RNA-Seq data set published by our group [23] for a more comprehensive study of early inflammation. GO term analysis on genes related to POI hallmarks revealed a decrease in genes associated with migration (e.g., leukocytes and neutrophils) and inflammation (e.g., immune and inflammatory response and leukocyte activation) after STX (Fig. 2F). Additionally, we also detected substantial alterations in gene clusters related to changes in the ENS, previously shown to be upregulated at IM3h in our *RiboTag* mice (Additional file 2: Fig. S2B), suggesting a direct effect of denervation on enteric glial reactivity and communication during gut inflammation (Additional file 2: Fig. S2C).

Consequently, we used the “acute enteric gliosis” GO term to investigate the transcriptional status of enteric glia and affected tissue after STX. While only a minority of genes were altered between naïve mice with and without 6-OHDA treatment, strong differences were observed in the majority of enteric gliosis genes at IM3h following STX, highlighting SNS involvement in the development of acute post-operative enteric gliosis (Fig. 2G).

These data suggest that increased sympathetic inputs, known to start simultaneously with surgery, immediately trigger enteric glial reactivity and modulate inflammatory and migratory gene expression.

### Sympathetic innervation triggers enteric glial reactivity already in the absence of intestinal manipulation

**Fig. 3:**
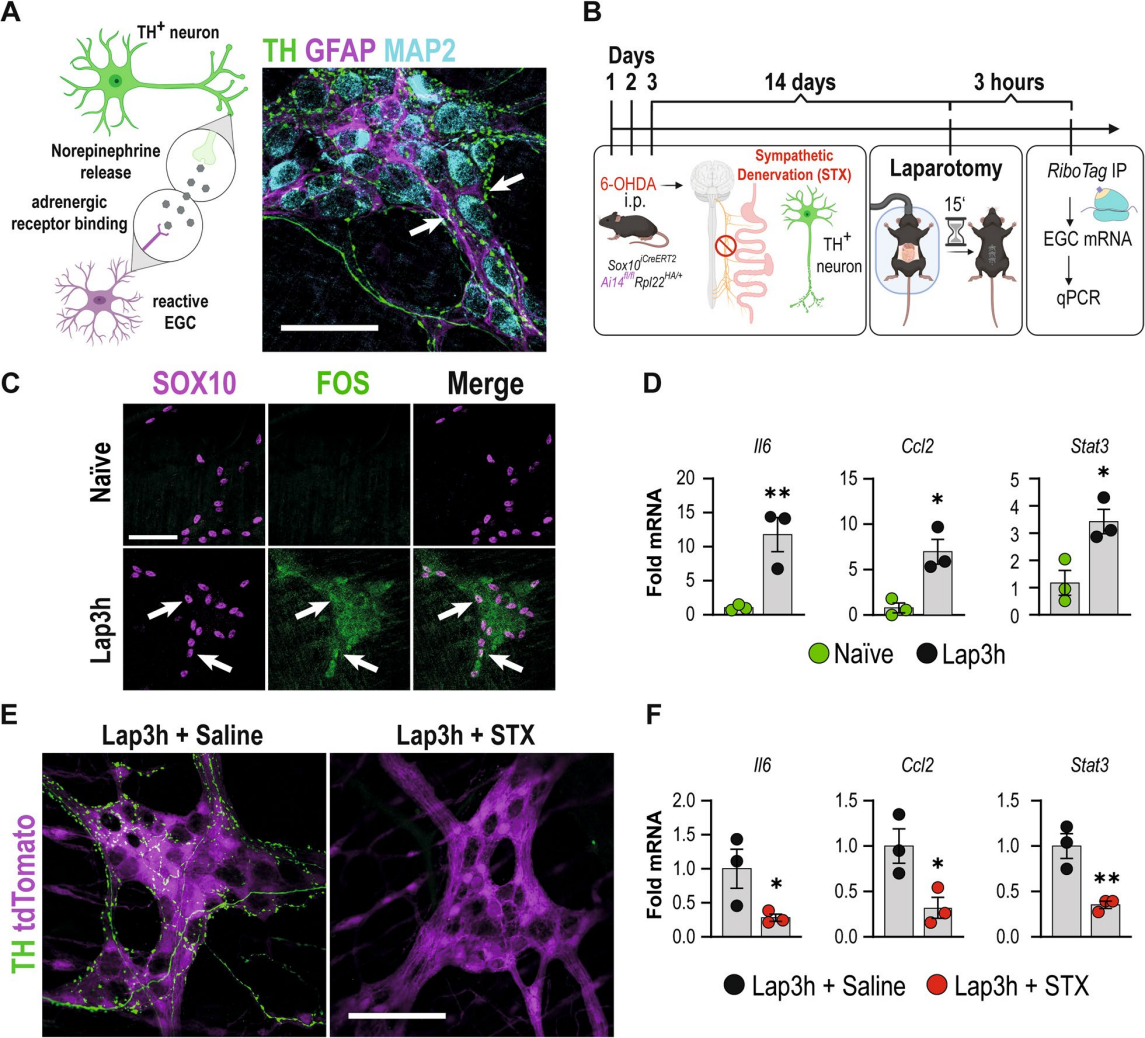
Enteric glia react before overt inflammation by receiving cues from the sympathetic nervous system. **A** Illustration of our hypothesis of TH^+^ neuron released NE triggering enteric glial activation and confocal images of immunohistological staining of sympathetic nerve fibers (TH, green), enteric neurons (MAP2; light blue), and enteric glia (GFAP, magenta). Arrows indicate TH^+^ fibers innervating the *ME*. Scale bar (50 µm). **B** Schematic description of chemical sympathetic denervation of *Sox10*^*iCreERT2*^*/Rpl22*^*HA/*+^ mice with three consecutive intraperitoneal 6-OHDA injections (days 1–3). After 14 days, the mice underwent Lap. *ME* was isolated three hours later (Lap3h), processed according to the RiboTag approach, and analyzed by qPCR. **C** Confocal images of immunohistological stainings of SOX10 (magenta) and FOS (green) expression in whole mounts of naïve and Lap3h small bowel *ME*. (n = 3 animals per condition). Arrows indicate FOS^+^ SOX10^+ ^enteric glia. Scale bar (100 µm). **D** qPCR analysis showing fold changes of mRNA levels (mean ± SEM) of *Sox10*^*iCreERT2*^*/Rpl22*^*HA/*+^ *RiboTag* mRNA from naïve and Lap3h mice for cytokines (*Ccl2, Il6*) and an early response marker (Stat3) (2^−ΔΔCT^, *18S/Tubb4*, Naïve; n = 3 animals per condition; Student’s t-test, * < 0.05, ** < 0.01). **(E)** Confocal images of immunohistological stainings of TH (green) and endogenous SOX10-tdTomato (magenta) expression in whole mounts of Lap3h + Saline and Lap3h + STX small bowel *ME*. (n = 3 animals per condition). Scale bar (100 µm). **F** qPCR analysis showing fold changes of mRNA levels (mean ± SEM) of *Sox10*^*iCreERT2*^*/Rpl22*^*HA/*+^ *RiboTag* mRNA from Lap3h + Saline and Lap3h + STX mice for cytokines (*Ccl2, Il6*), and an early response marker (Stat3) (2^−ΔΔCT^, *Tubb4/Actb/PGK/GAPDH*, Naïve; n = 3 animals per condition; Student’s t-test, * < 0.05, ** < 0.01)

Earlier work showed that inflammation within the small intestinal *ME* already occurs upon abdominal incision without surgical manipulation of the visceral organs. As intestinal sympathetic over-activation is known to start with the abdominal incision [42], we speculated that sympathetic projections of TH^+^ neurons, innervating the *ME,* might also signal to enteric glia by an immediate release of NE after the abdominal incision, thereby directly activating enteric glia. Confocal microscopy revealed close proximity of TH^+^ nerve fibers with MAP^+^ neurons and GFAP^+^ enteric glia in myenteric ganglia (Fig. 3A), anatomically supporting the idea of a direct SNS to enteric glia communication. To test this hypothesis, we subjected *Sox10*^*iCreERT2*^* RiboTag* mice to a laparotomy without eventration or manipulation of the intestine (Fig. 3B). FOS expression, an early cellular activation marker, and a representative enteric gliosis gene was not detected in SOX10^+^ enteric glia in naïve mice. At the same time, laparotomy elicited FOS immunoreactivity in myenteric ganglia, including SOX10^+^ expressing enteric glia (arrows, Fig. 3C). Notably, intestinal manipulation aggravated FOS immunoreactivity in the *ME*, particularly in enteric glia (marked by arrows, Additional file 2: Fig. S3A). These data show that an enteric glial activation already occurs immediately after the surgical incision without surgical manipulation of the intestine, while the latter is a potent enhancer of enteric glial reactivity. To confirm that a laparotomy is sufficient to trigger acute enteric gliosis genes, we performed a laparotomy in *Sox10*^*iCreERT2*^* RiboTag* mice. We detected the induction of representative acute enteric gliosis genes (i.e., *Il6*, *Ccl2,* and *Stat3*) compared to naïve animals (Fig. 3D). Consequently, we used 6-OHDA in *RiboTag* mice to test whether sympathetic innervation triggers the laparotomy-induced enteric gliosis genes. Analogous to our wildtype mice, 6-OHDA depleted the TH^+^ neuronal processes in *RiboTag* mice and left the glial network intact (Fig. 3E). In line with our hypothesis, *Il6*, *Ccl2*, and *Stat3* gene expression levels were reduced in enteric glia in STX mice compared to saline-treated controls (Fig. 3F). Notably, cellular and functional POI hallmarks also occur in laparotomized mice, although to a lesser extent, with a distinct increase in infiltrating myeloperoxidase^+^ cells (Additional file 2: Fig. S3B) and reduced gastrointestinal motility compared to naïve mice (Additional file 2: Fig. S3C).

Overall, these data uncovered that the SNS contributes to the induction of genes involved in acute enteric glial reactivity already after the initial abdominal incision, which is further aggravated by mechanical manipulation of the intestine.

### NE triggers acute enteric gliosis and activates enteric glia via β-adrenergic receptors

**Fig. 4:**
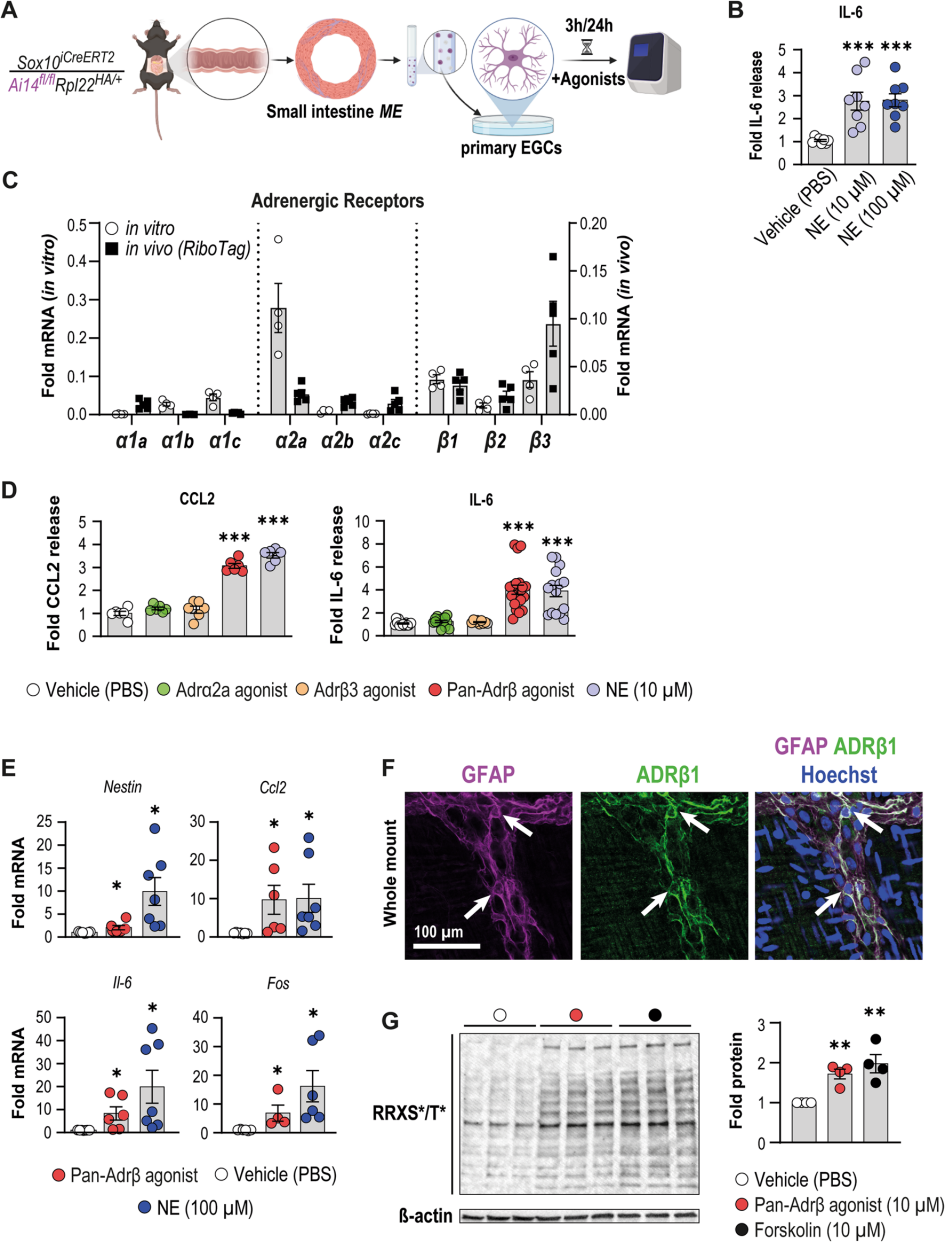
NE triggers acute primary enteric gliosis and glial reactivity via β-adrenergic receptors. **A** Schematic of primary EGC cultures from *Sox10*^*iCreERT2*^*/Rpl22*^*HA/*+^*/Ai14*^*fl/fl*^ mice. **B** ELISA analysis for IL-6 (mean ± SEM) from conditioned medium of cultured EGCs after stimulation with vehicle (PBS) or NE (3 h; 10 µM, 100 µM) (n = 8 distinct cell culture wells per condition; multiple unpaired t-tests, comparison to vehicle, *** < 0.001). **C** qPCR analysis (mean ± SEM) of *Sox10*^*iCreERT2*^*/Rpl22*^*HA/*+^ *RiboTag* mRNA and RNA from cultured primary EGCs for different adrenergic receptors (2^−ΔΔCT^, *Tubb4/Actb/PGK/GAPDH*, n = 5 distinct cell culture wells per condition). **D** ELISA analysis for IL-6 and CCL2 (mean ± SEM) from conditioned medium of cultured primary EGCs after stimulation (3 h) with vehicle (PBS), adrenergic agonists (against: α2a, β3, pan-β (isoprenaline)), and NE (all 10 µM); CCL2: n = 6 distinct cell culture wells per condition; IL-6: n = 9–21 distinct cell culture wells per condition; multiple unpaired t-tests, comparison to vehicle, *** < 0.001). **E** qPCR analysis (mean ± SEM) of cultured primary EGCs for acute gliosis genes after vehicle (PBS), isoprenaline (100 µM), or NE (100 µM) treatment (2^−ΔΔCT^, *18S*, Naïve; n = 4–7 distinct cell culture wells per condition; multiple unpaired t-tests, comparison to vehicle, * < 0.05). **F** Immunohistochemistry of cryo-embedded intestinal specimens stained for GFAP^+ ^enteric glia (magenta) and ADRβ1 (green) in the *ME*; Hoechst was used to detect cell nuclei (white). Arrows indicate double-positive cells. Scale bar (50 µm). **G** Western blot and corresponding densitometry (mean ± SEM) of lysates of primary EGC cultures treated with vehicle, isoprenaline (10 µM), or forskolin (10 µM) stained for phosphorylated cAMP-dependent protein kinase (pPKA) (multiple bands) and β-actin (~ 42 kDa) as loading control (n = 4 cultures from 4 different animals treated with the compounds or vehicle; representative blot shows three technical replicates of one biological repeat per condition); multiple unpaired t-tests, comparison to vehicle, ** < 0.01)

As ablation of TH^+^ neurons led to a reduced acute enteric gliosis, we next assessed whether NE, the principal postganglionic sympathetic neurotransmitter, induces enteric glial reactivity. Therefore, we treated primary murine EGC cultures from small bowel *ME* specimens (Fig. 4A) with NE, which caused an almost threefold increase in IL-6 protein release after 3 h (Fig. 4B) that further increased in a dose-dependent manner 24 h after NE treatment (Additional file 2: Fig. S4B). In addition, NE also triggered *Gfap*, *Nestin*, *Stat3, Fos*, and *Ccl2* gene expression 3 h post-treatment (Additional file 2: Fig. S4C). Notably, the induction of these reactive glia marker genes was transient and disappeared after 24 h (Additional file 2: Fig. S4C), resembling the immediate in vivo enteric glial activation pattern seen in laparotomized and intestinally manipulated mice.

Since NE signals through various adrenergic receptors, we next aimed to characterize their expression profile in primary EGCs to elucidate possible receptors involved in acute enteric gliosis induction. RNA samples of naïve *Sox10*^*iCreERT2*^* RiboTag* mice and cultured primary EGCs revealed α2a adrenergic receptor (AR) and the three β-ARs β3 > β1 > β2 as the highest expressed on primary EGCs in vitro and in vivo (Fig. 4C). However, immunocytochemistry of α2a-AR showed no expression in primary EGCs, while it was strongly expressed in non-glial cells in vitro in primary EGC cultures (Additional file 2: Fig. S4D). We next stimulated the three β-ARs and the α2a-AR (as an anticipated negative control) with selective adrenergic agonists in cultured primary EGCs. We analyzed IL-6 and CCL2 release by ELISA and other representative enteric gliosis genes by qPCR. The pan-β-AR agonist isoprenaline elicited IL-6 and CCL2 protein release to the same extent as NE, while neither the α2a-AR-specific agonist (guanfacine) nor the β3-AR-specific agonist (CL-316243) triggered any release (Fig. 4D). Accordingly, isoprenaline and NE significantly induced *Ccl2, Il6, Nestin,* and *Fos* gene expression (Fig. 4E). By immunohistochemistry of *ME* whole-mounts and intestinal cross-sections, we detected β1-AR in GFAP^+^ ganglia (Fig. 4F**,** Additional file 2: Fig. S4E) and verified the signal with an IgG control (Additional file 2: Fig. S4F). Furthermore, we wanted to investigate the activation of adrenergic signaling cascades upon treatment with isoprenaline. We performed SDS-PAGE and western blotting in combination with an antibody specifically binding to the consensus phosphorylation sequence (RRXS*/T*) of all targets of the activated/phosphorylated form of cAMP-dependent protein kinase A (Fig. 4G), a primary signaling molecule for the adrenergic pathway. Here, we observed a significant increase in the amount of phosphorylated targets after treatment of primary EGCs with 10 µM isoprenaline for 1 h (Fig. 4G), comparable to the activation induced by our positive control treatment forskolin (10 µM).

Since the pan-β-AR agonist isoprenaline, but not the β3-AR-specific agonist, induced a molecular enteric gliosis signature and activated downstream kinases in primary EGCs, we deduced that NE released by the SNS after the onset of surgery activates β1- or β2-AR signaling in enteric glia.

### Ex vivo and optogenetic activation of adrenergic downstream signaling triggers enteric glial reactivity

**Fig. 5:**
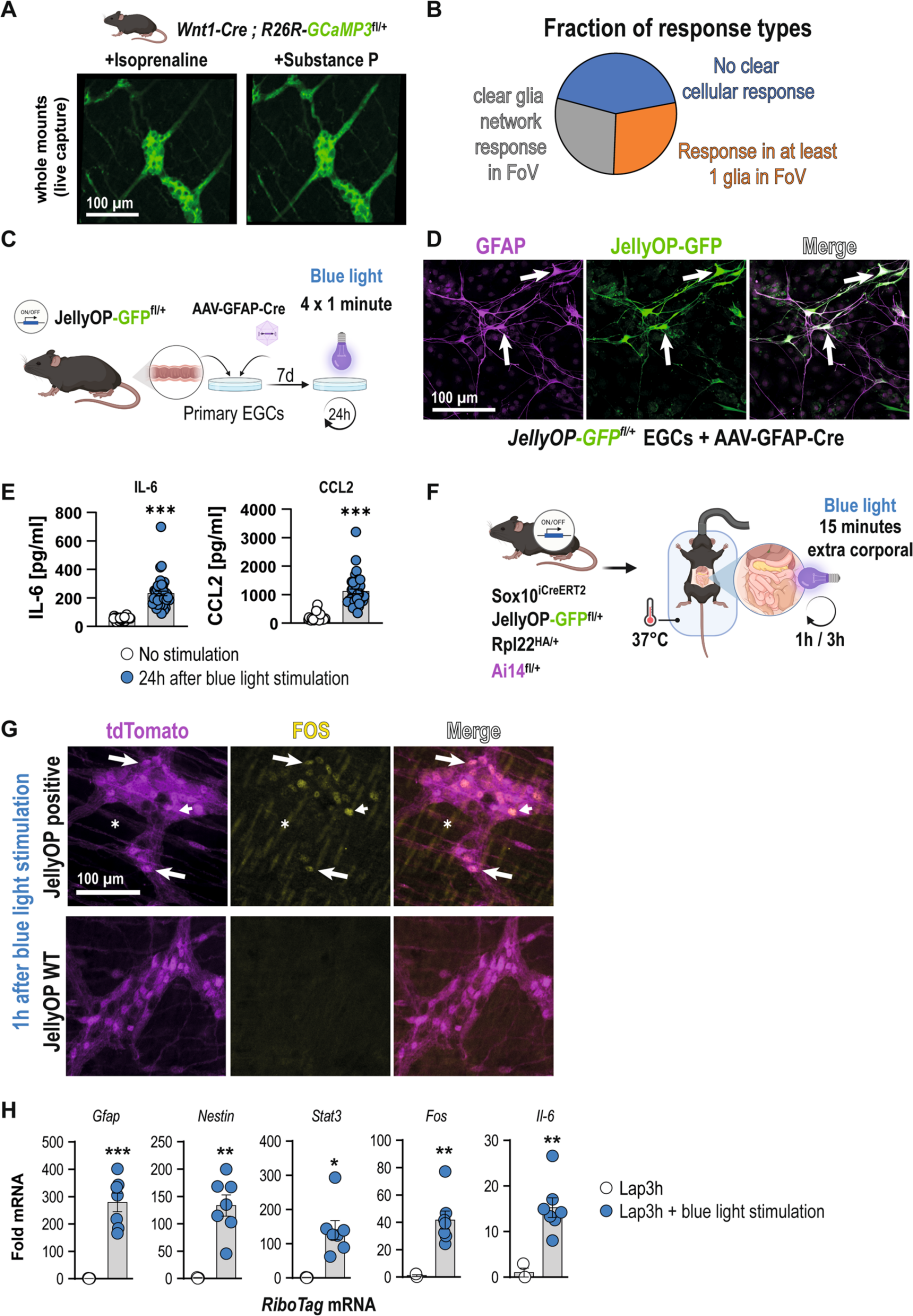
Ex vivo and optogenetic activation of adrenergic downstream signaling triggers enteric glial reactivity. **A** Image frames taken during the acute exposure to isoprenaline (left panel, 10 µM) or Substance P (right panel, 10 µM) in *Wnt1-Cre; R26R-GCaMP3*^*fl/fl*^ mice. **B** Pie plot depicting the response types observed in the 14 ganglia (n = 4 mice). **C** Schematic of the primary culture preparation, viral transfection, and in vitro activation process of *JellyOP*^*fll*+^ mice with blue light. **D** Confocal images of the *JellyOP-GFP* construct in tdTomato-Sox10^+^ cells seven days after transfection with the AAV-GFAP-Cre. Arrows indicate GFP^+^/GFAP^+^ glia. Scale bar (100 µm). **E** ELISA analysis for IL-6 and CCL2 (mean ± SEM) from conditioned medium of cultured primary EGCs from *JellyOP*^*fl/*+^ mice transfected with an AAV-GFAP-Cre after stimulation with blue light or without stimulation (n = 24–42 separately transfected wells from two distinct isolations; Student's t-test, *** < 0.001). **F** Schematic of the in vivo activation process of *Sox10*^*iCreERT2*^*/Rpl22*^*HA/*+^*/Ai14*^*fl/*+^*/JellyOP*^*fl/*+^ mice. **G** Confocal images of IHC for FOS (green) and SOX10 (magenta) in whole mounts of *Sox10*^*iCreERT2*^*/Rpl22*^*HA/*+^*/Ai14*^*fl/*+^*/JellyOP*^*fl/*+^ or *Sox10*^*iCreERT2*^*/Rpl22*^*HA/*+^*/Ai14*^*fl/*+^*/JellyOP*^+*/*+^ *mice* 1 h after stimulation with blue light. Arrows indicate FOS-positive SOX10 glia. Scale bar (100 µm). **H** qPCR analysis of mRNA (mean ± SEM) from *Sox10*^*iCreERT2*^*/Rpl22*^*HA/*+^*/Ai14*^*fl/*+^*/JellyOP*^*fl/*+*l*^ mice or *Sox10*^*iCreERT2*^*/Rpl22*^*HA/*+^*/Ai14*^*fl/*+^*/JellyOP*^+*/*+^ *mice* 3 h after optogenetic activation and laparotomy for gliosis hallmark genes (2^−ΔΔCT^, *18S*, *JellyOP*-negative animals, n = 3–7 animals per genotype; Student’s t-test, *** < 0.001, * < 0.05)

To better understand the in vivo activation of enteric glia by beta-adrenergic signaling in living tissue and in vivo, we utilized several additional models. We first assessed glial responses in an ex vivo approach wherein we used ileal tissue from *Wnt1-Cre;R26R-GCaMP3* mice to test whether isoprenaline (10 µM) would elicit a glial Ca^2+^ response in the myenteric ganglia. Using a local perfusion system, isoprenaline was applied directly onto the ganglion (Fig. 5A; Additional file 3A and B). Additionally, using the same multi-barrel perfusion tip, Substance P (10 µM) was used to identify the glia cell network [36], and high K^+^ to identify the neurons (Additional file 2: Fig. S5A). Fourteen ganglia were imaged in separate preparations, and although the responses were variable between recordings and mice, some clear glial cell network activation was seen in a fraction of the recordings (4/14). In contrast, in others (4/14), at least one enteric glial cell in the field of view (FOV) responded (Fig. 5B). In the six other recordings, isoprenaline induced a small contraction, and no cellular (neither neuronal nor glial) Ca^2+^ response could be detected. The relative amplitude of those glial cells that were responding to isoprenaline showed a small delay in reaction time with half the amplitude (47 ± 6%) of Substance P used to identify them as glial cells (n = 45 cells; Additional file 2: Fig. S5B). Therefore, we assume that β-AR-stimulated enteric glia acquire a reactive state and can react with distinct Ca^2+^ responses.

Furthermore, we attempted to expand on our denervation experiments by pharmacological manipulation of adrenergic signaling. Therefore, we injected reserpine, a long-lasting inhibitor of monoamines and subjected these mice, as well as mice without reserpine injection, to our laparotomy model (Additional file 2: Fig. S5D). Since sympathetic denervation led to reduced gliosis, we expected the injection of reserpine to mimic this effect, but could not detect significant changes between the laparotomy groups with and without reserpine (Additional file 2: Fig. S5E). We next tested if a chemical increase in NE release induces a POI-like phenotype. Therefore, we applied tyramine, an indirect sympathomimetic compound facilitating catecholamine release [31] (Additional file 2: Fig. S5F). However, no significant changes were observed between the tyramine and control group with the chosen administration scheme (Additional file 2: Fig. S5G).

Finally, we addressed the question if β-adrenergic signaling can indeed directly trigger enteric glial reactivity by utilization of an optogenetic tool, the Jellyfish-Opsin (*JellyOP)*-construct, which enables selective optogenetic activation of the adrenergic G_s_ signaling by blue light stimulation [43]. For cell-type specific expression of JellyOP and GFP after Cre-mediated excision of a floxed stop cassette, a new mouse line (*JellyOP-GFP*^*fl/*+^) was generated by CRISPR/Cas9 mediated gene-editing of the Rosa26 locus (30). Starting with an in vitro approach, primary EGCs from *JellyOP-GFP*^*fl/*+^ mice were transfected with an AAV-GFAP-Cre during their seven days of maturation (Fig. 5C) to generate EGFP-expressing *JellyOP* EGCs. These *JellyOP* EGCs were blue light stimulated and analyzed 24 h later for GFAP and GFP expression as well as IL-6 and CCL2 expression by ELISA. Confocal microscopy revealed a strong GFP expression in GFAP^+^ enteric glia, indicating successful viral transfection and Cre-activity (Fig. 5D) in primary EGCs. In line with the previous isoprenaline treatment, we detected a significant increase in IL-6 and CCL2 release after blue light stimulation (Fig. 5E), proving that optogenetic activation of adrenergic (G_s_) downstream signaling in EGCs causes an inflammatory glial phenotype.

Next, we performed an in vivo study with the *JellyOP* system by generating a specific mouse line that utilizes our *Sox10*^*iCreERT2*^* RiboTag* approach in conjunction with the *JellyOP-GFP*^*fl/*+^ mice. The resulting glial *JellyOP RiboTag* mice enable the direct optogenetic activation of enteric glial-restricted adrenergic downstream signaling and assessment of their transcriptome. Immunohistochemistry confirmed the successful expression of the *JellyOP*-GFP construct exclusively in enteric glia of the small intestinal *ME* (Additional file 2:: Fig. S6A). To validate an immediate glial response upon the beginning of surgery, our glial *JellyOP* mice and *JellyOP*-negative litter mates were laparotomized, the small bowel was carefully eventrated, and the jejunum and ileum were illuminated with blue light (Fig. 5F). Immunohistochemistry revealed a strong FOS expression already 1 h after blue light stimulation (Fig. 5G) in glial *JellyOP*-positive animals, while reactivity was almost absent in mice lacking the *JellyOP*.

Interestingly, smooth muscle cell nuclei, identified by their longitudinal shape (Fig. 5G, asteriks) and SOX10-negative ganglionic cells, likely enteric neurons (Fig. 5G, arrowheads), also stained positive for FOS, indicating that these cells become activated due to the glial-specific activation of G_s_ signaling. Similar FOS histology results were obtained 3 h after stimulation (Additional file 2: Fig. S6B) showing even more cells activated, which is comparable to IM3h whole mount specimens of *JellyOP RiboTag* mice (Additional file 2: Fig. S6C). Strikingly, when we compared blue light-treated glial *JellyOP*-positive and *JellyOP*-negative *RiboTag* mice by qPCR analysis, a strong upregulation of several gliosis panel genes, e.g., *Il6, Fos, Stat3, Gfap,* and *Nestin*, were detected (Fig. 5H). Of note, *Ccl2* expression was only detectable in 2 of 3 glial *JellyOP*-negative *RiboTag* mice, but it was highly expressed in *JellyOP*-positive litter mates (data not shown). To ensure that JellyOP RiboTag mice with a mixed CD1/BL6 background develop a classical POI, we performed IM and analyzed general disease hallmarks. IM evoked a distinct increase in leukocyte infiltration in JellyOP RiboTag mice (Additional file 2: Fig. S6D, SE) and a reduction of gastrointestinal motility (Additional file 2: Fig. S6H) 24 h after surgery compared to naïve littermates. These changes were similar to those observed in mice on a pure BL6 background. Moreover, to validate the gliosis state, we analyzed glial cell proliferation and detected a comparable increase in Ki67 + /SOX10 + cells 24 h after IM (Additional file 2: Fig. S6F, G) as seen before in BL6 mice (Fig. 1B; [5]), solidifying the use of this strain even without backcrossing to a pure BL6 background.

In conclusion, our data show that abdominal surgery immediately induces a transient reactive enteric glia phenotype in the small intestine *ME*. Surgery-induced SNS activity, confirmed by optogenetically induced G_S_ and chemically induced β-adrenergic stimulation of enteric glia, can trigger this phenotype directly via β1/2-AR signaling immediately after the initial surgical incision. Selective antagonism of these receptors might be a potential future target to modulate enteric glial reactivity and their functional consequences in immune-driven intestinal disorders.

## Discussion

**Fig. 6:**
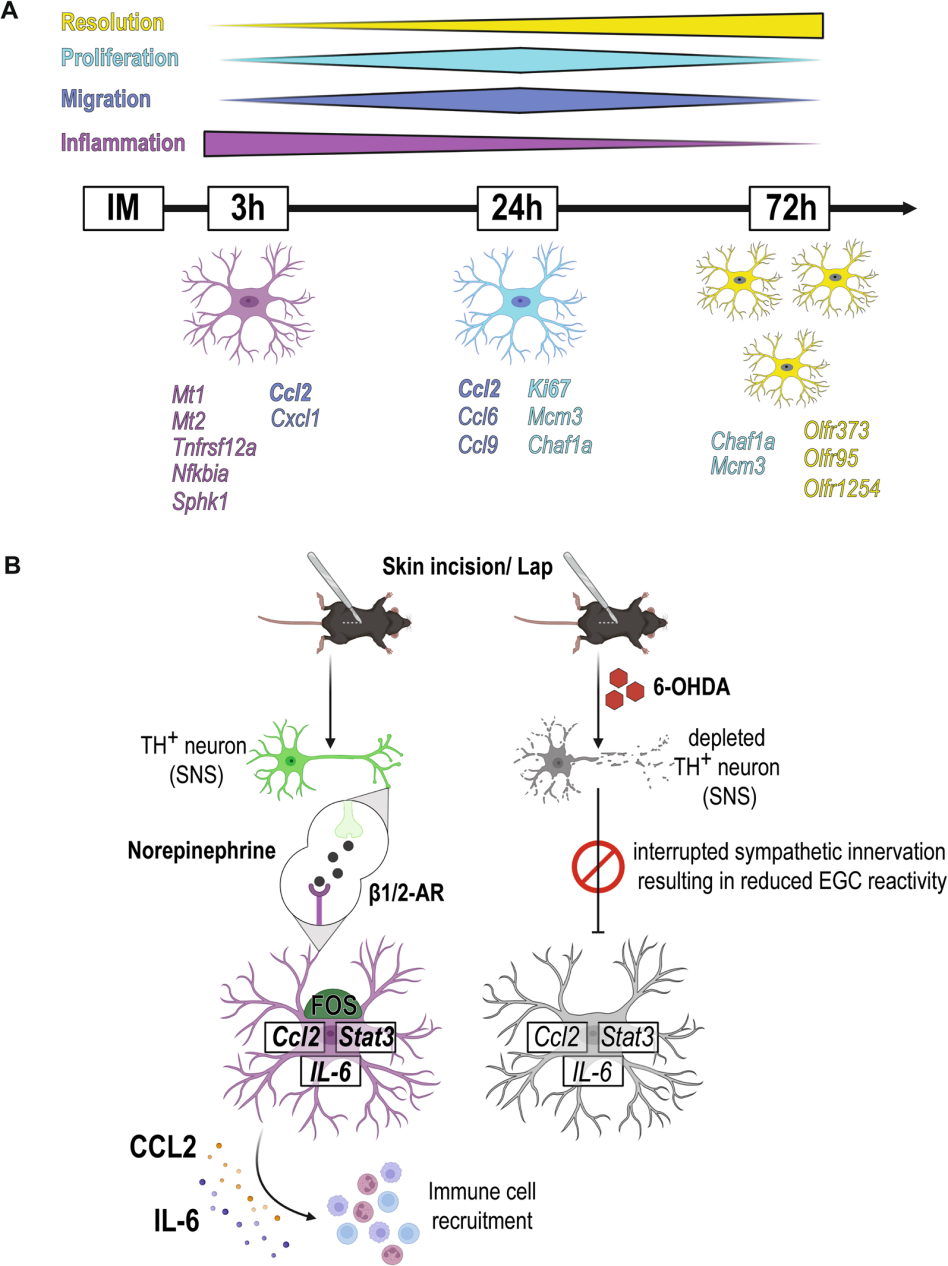
β-adrenergic signaling in enteric glia triggers enteric glial reactivity and modulates intestinal inflammation in POI. **A** Graphical abstract of the longitudinal analysis of enteric glia after intestinal manipulation (IM) showing a distinct separation into three stages defined by specific hallmarks. Initial reaction (IM3h, magenta) includes an immediate inflammatory reaction and the shaping of the intestinal environment in concert with the initiation of cell migration (blue). This migratory phenotype manifests further 24 h after IM and is accompanied by elevated proliferation (light blue). Finally, inflammatory reactions of enteric glia continuously taper off and are gradually replaced by resolution-related genes (yellow) that initiate a return to the regular intestinal environment. **B** Graphical abstract of SNS activation of enteric glia. Skin incision and laparotomy (in the absence of IM) lead to immediate activation of the SNS, which triggers enteric glial reactivity in the small bowel *ME*. Sympathetic nerve endings in the *ME* release NE, which binds to adrenergic receptors β1 or β2 on myenteric enteric glia. Enteric glia subsequently become reactive (FOS, *Stat3*) and induce upregulation and release of inflammatory mediators (CCL2, IL-6) that in turn modulate immune cells. Chemical disruption of the sympathetic innervation reduces the reactive enteric glia phenotype and the postsurgical inflammatory response

Enteric glia are an immuno-active cell type involved in intestinal homeostasis that act on the level of local tissue inflammation. They communicate with neurons and immune cells [44] and are discussed as potential targets in treating or preventing immune-driven intestinal disorders [45]. In our previous studies, we have shown that enteric glia trigger a tissue-related inflammatory state, termed “enteric gliosis”, triggered amongst others by immune-mediators IL-1 [41] and ATP [5], finally resulting in postoperative *ME* inflammation and POI. However, the individual molecular mechanism in this disease-specific acute enteric gliosis state and the cellular response of enteric glia remained elusive. Herein, we now selectively analyzed enteric glia-specific transcriptional responses by the *RiboTag* approach [46], a tool to isolate actively transcribed mRNA selectively from a target cell population in the tissue of interest [28]. This technique delivered longitudinal transcriptional data of enteric glia in all relevant POI stages, thereby depicting a compelling transition of enteric glial reactivity within different phases of POI. This transition is structured in three stages: an early transcriptional switch of enteric glia into an inflammatory type, a stage defined by an active release of chemotactic factors (migratory), and an increase in proliferation markers (proliferation), and ultimately a state of tissue regeneration and inflammatory resolution (resolution) (Fig. 6A), which are remarkably similar to the classical POI disease development [25]. Notably, gene expression patterns do not significantly overlap between these phases.

Our Ribotag approach generated a list of 243 early induced and actively transcribed genes that we compiled into a novel GO term, "acute enteric gliosis". We hypothesize that reactive enteric glia are a crucial starting point for the subsequent inflammatory changes in cellular and molecular composition in the *ME* environment [47], thereby resembling a critical time point for potential intervention strategies involving enteric glia. Some of the genes immediately induced in enteric glia by IM confirmed our previous studies, e.g., induction of IL-1 and purinergic target genes [5, 41]. In these studies, we showed that ATP via the P2X2 receptor leads to enteric gliosis and cytokine release [5], and IL-1 signaling evokes enteric glial reactivity that leads to *ME* macrophage recruitment [41]. However, our new findings demonstrate that early enteric glial activation emerges as a multifactorial process requiring a variety of stimuli. Furthermore, other factors that are also well-known markers for glial reactivity in the CNS, including *Ccl2 *[48], *Cxcl1 *[48, 49], *Mt2* [50]*, Nes* [9, 49], and *Il6* [48], can now be directly attributed to enteric glial reactivity in the acutely inflamed small bowel *ME*. Notably, some of these molecules, e.g., CCL2 [51], IL-6 [52], and CXCL1 [53], have already been analyzed for their molecular or immune-modulatory function in POI. Another important chemokine-induced in enteric glia during POI is CXCL10, which was recently implicated during acute infection [11] and is also known in context with CNS gliosis [49]. Moreover, metallothioneins *Mt1* and *Mt2*, essential regulators of oxidative stress in reactive astrocytes of MS patients [54], were highly induced during early inflammation. In addition to the prominently expressed chemotactic factors and immune mediators, we detected others that are novel in this context. For instance, genes of the EGF-family, such as *betacellulin* (*Btc*), previously indicated in ileal growth and homeostasis [55] during health, and *amphiregulin* (*Areg*), implicated in intestinal homeostasis during colitis [56], was induced (*Btc*) or even de novo expressed (*Areg*) upon trauma. Regarding gliosis, we detected an upregulation of *Ier5l* and *Ifrd1*, two genes of the “immediate-early gene” group that regulate cell growth and immune function [57] and include other factors., e.g., *Fos*, *Egr1/2*, *Nr4a1/2*, *Jun*, *Atf3,* and *Fosl*, all strongly induced upon IM. Nevertheless, most (thirty-six) of the top 50 induced genes have inflammatory and chemotactic functions supporting the central role of enteric glia as modulators and initiators of acute inflammatory responses in the gut [1, 44].

The transcriptional profiling suggests the enormous plasticity of enteric glia in acute inflammation. The high enteric glial plasticity was recently analyzed and discussed by Guyer et al., substantiating the enteric glia potential to manage multiple gut functions [58]. Within the progression of POI, the enteric glia phenotype switches from the initial inflammatory phenotype over a proliferation state towards a so far undefined phenotype. In the proliferation state, we detected a substantial increase in Ki67^+^/SOX10^+^ enteric glia and attributed this cell cycle activation to the POI-related enteric gliosis phenotype. Our countings of the total number of SOX10^+^ enteric glia could verify the increase in cell numbers 72 h after surgery. Interestingly, recent publications also discuss additional functions of Ki67, such as its role in cell cycle arrest and/or cell synchronization [59, 60]. These functions may play a role in the phenotypic switch of enteric glia during intestinal inflammation in POI.

In the late phase, enteric glia express several olfactory receptors (e.g., *Olfr373* and *Olfr95*) recently shown as “resolution genes” of intestinal inflammation [40, 61] and discussed as potential therapeutic targets to control inflammation and healing [62]. Other OLFRs like OLFR544 [61] or OLFR78 [40] also control gut inflammation, and some were shown to detect and signal to short-chain fatty acids, e.g., butyrate and propionate, produced by luminal bacteria, which act on multiple levels to control intestinal health and disease [63].

Overall, the first part of our study delivers new molecular insight into enteric glial plasticity during intestinal inflammation. While glial reactivity is highly tissue, trigger and disease state dependent, resulting in a different outcome for the cells and their surrounding environment, these distinct molecular signatures will be a valuable starting point for other research. Certainly, enteric glial reactivity varies with treatment conditions in different disease models, including inflammatory bowel diseases [15, 64] or colorectal cancer [65].

Any abdominal surgery starts with the incision of the skin and the abdominal wall before any surgical manipulation of the visceral organs occurs. More than two decades ago, Kalff et al. found that the initial steps of a laparotomy already triggered *ME* inflammation [42]. Accordingly, sympathetic reflexes and activation of the SNS are known to occur early in surgery. The SNS connects to a variety of cells, including enteric ganglia [27] and intestinal macrophages, wherein they exert β2-AR-mediated immune-modulatory function in infectious [20] and immune-driven diseases [21]. Furthermore, sympathetic neurotransmitters, such as NE, regulate motility [66] and control immune cell migration during inflammatory events. As our findings revealed a particular role of enteric glial reactivity within the immediate postoperative phase, when the SNS becomes activated by surgery, and the principal sympathetic neurotransmitter NE is known to modulate the postoperative immune response in POI [23], we were wondering if sympathetic pathways might trigger the early activation of enteric glia.

Indeed, enteric glia responded to the initial bowel wall incision before the actual surgical manipulation of the intestine began. Enteric glia released cytokines such as *Il6* and *Ccl2*, and ablation of SNS nerve fibers ameliorated the acute postsurgical enteric gliosis by reducing cytokine production and early response gene transcription. This diminished glial reactivity was accompanied by a decrease in several POI hallmarks, such as migration and inflammatory response (Fig. 6B).

Further evidence about functional alterations of glial cells to adrenergic stimulation comes from observations in the CNS [67]. The findings of Tong et al. supported the immunomodulatory role of sympathetic inputs, as 6-OHDA-mediated ablations of sympathetic nerves resulted in diminished activation of spinal cord glia [68]. Another study showed that the blockage of β-AR prior to cytotoxic insults to the spinal cord reduced reactive gliosis [69]. SNS neurotransmitters can elicit pro- or anti-inflammatory cytokine release depending on the tissue environment, concentration, and AR binding [70]. In the CNS, for example, NE reduced astrocyte swelling after spinal cord injury [71] and elicited neuroprotective effects in H_2_O_2_-treated neuron/glia co-cultures [72]. In contrast, preconditioning with NE before ischemic injury exacerbated reactive gliosis [73], and β-2AR agonist treatment increased IL-6 expression after TNFα-induced inflammation in vivo and in vitro [74]. Another pathway triggered by adrenergic activation, partially glimpsed in our experiments, might be the modulation of calcium signaling, which was recently observed in vitro in HEK cells [75] and in vivo in cardiac myocytes [76]. Moreover, adrenergic agonists evoked calcium changes in cultured astrocytes and astrocyte networks in hippocampal slices [77], further prompting future research into this interaction in the gut.

Interestingly, SNS action during inflammation can have opposing effects. While chemical denervation showed beneficial effects during acute inflammation in POI [23] it had adverse effects in chronic intestinal inflammation [78, 79] and mice suffering from physical stress together with colitis [80]. Supporting the beneficial effect of the SNS in chronic inflammation, a recent study by Schiller et al. showed that repeated optogenetic stimulation of TH^+^ fibers attenuated symptoms of DSS colitis by reducing immune cell abundance [81]. In IBD patients, the use of β-blockers is associated with an increased relapse risk [82]. Based on the spontaneous increase in glial calcium signaling upon β-adrenergic stimulation, the immediate induction of reactive glia marker genes after blue light activation of a downstream G_s_ cascade that mimics β-adrenergic pathways [43] in our *JellyOP* experiments, and the observation that a chemical sympathetic denervation improves symptoms of POI [23], we believe that sympathetic actions in acute inflammatory conditions are rather detrimental. To this end, we expected a more profound insight from our in vivo experiments with reserpine and tyramine application. However, the tested conditions, adapted from previous publications that used these drugs in vivo [31, 32]*,* failed to show changes in POI symptoms and transcriptional glial reactivity compared to the control groups. The lack of strong pharmacological in vivo studies for both compounds, particularly in gastrointestinal physiology and immunology, thus warrants a need for further studies. These should include in-depth pharmacological analyses, comparisons of different administration routes, use of different drug concentrations, time points, and durations before a clear statement about the mode of action of reserpine and tyramine in sympathetic in vivo modulation within the intestine can be claimed.

Our study emphasizes that adrenergic signaling is complex and exerts distinct roles in different cell types, organs, and diseases to control cellular reactivity during inflammation. This knowledge might also be of clinical relevance, e.g., in the application of adrenergic blockers in patients suffering or expected to suffer from acute or chronic intestinal inflammatory diseases. Depending on the nature of the underlying disease, adrenergic blockers can evoke beneficial or detrimental health effects.

As chemical denervation ameliorated postoperative glial reactivity and reduced acute enteric gliosis, we propose SNS-based neurotransmitter release acting on enteric glia as the mechanism of their early activation, a state aggravated by the manipulation of the intestine and additional signaling cascades such as ATP and IL1 signaling. Imura et al. provided supporting evidence of an adrenergic signaling-induced reactive astrocyte phenotype upon stimulation with isoprenaline [83] accompanied by an increased β-AR expression in reactive astrocytes in vivo. Our findings suggest β1- or β2-AR as the relevant receptors to propagate SNS-stimulated inflammatory changes in enteric glia, as the pan-β-AR agonist isoprenaline, but not β3-AR agonism evoked a cytokine release. Applying β2-AR agonist salbutamol also ameliorated DSS-induced ulcerative colitis [84]. Interestingly, antagonists against β-ARs reduced both cardiac inflammation (metoprolol) [85] and ulcerative colitis (propranolol) [86]. Further studies utilizing additional β-AR-specific agonists (e.g., isoprenaline or salbutamol), antagonists (e.g., metoprolol and propranolol), or glial-specific AR-knockouts can help to decipher inflammation-driven diseases involving glial activation as part of their pathophysiology.

It should be noted that adrenergic signaling-induced enteric glial reactivity might not, per se*,* be a purely detrimental factor in inflammation. Enteric glia appear to be highly plastic during inflammation. While initial NE interaction aggravates the acute enteric glial reactivity, prolonged NE exposure, as it occurs during prolonged or chronic inflammation, might drive enteric glia to a beneficial phenotype and partially explain the positive influence of the SNS during those stages [78, 79, 84]. Moreover, the inflammatory environment changes during the disease course, altering the cellular and molecular composition and thus might also affect the responses to adrenergic signaling. For instance, in the presence of TNFα, NE binding to β-ARs inhibited further TNFα and downstream IL-6 secretion, while adrenergic signaling in the absence of TNFα directly stimulated IL-6 secretion [70]. Moreover, AR receptor expression is modulated during inflammation, previously shown for astrocytes [83], and in the intestine controlled by sympathetic inputs [23]. As inflammation can be accompanied by a later loss [70], the absence of feedback loops might also explain the differences in adrenergic immune-related responses between acute and chronic inflammation. Notably, differences in the adrenergic reactions might also occur within different locations of the same organ. While chronic intestinal disease models focus on the mucosal layer, the acute postoperative response to surgery is mainly limited to the *ME* [87], which comprises a different cellular composition and more distant localization to the luminal contents of the gut.

Overall, our study delivers an unknown longitudinal insight into enteric glial molecular responses and reactivity during different phases of an acute inflammation-driven intestinal disorder. We introduced sympathetic adrenergic signaling as a priming factor of enteric glial reactivity and a potential therapeutic target. These data are of important future value, as they not only present an interventional target to control inflammation but will also help to understand similarities and differences in enteric glial reactivity in other inflammation-driven diseases, such as IBD and gastrointestinal cancer development.

### Supplementary Information

**Additional file 1:** **Method S1.** RiboTag approach in the muscularis externa. **Method S2.** Construction of the *JellyOP* targeting vector for the mouse Rosa26 locus. **Method S3.** *JellyOP* animal creation. **Method S4.** Recombinant adeno-associated virus (rAAV) preparation. **Table S1.** Buffer and media. **Table S2.** Antibodies. **Table S3.** PCR primer. **Table S4.** Acute enteric gliosis GO-term.

**Additional file 2: Figure S1.** Characterization of POI in *Sox10*^*iCreERT2*^*/Rpl22*^*HA/*+^ mice. **Figure S2.** Sympathetic signaling is involved in enteric glia functions. **Figure S3.** Laparotomy affects molecular functions. **Figure S4.** Effect of NE and tissue expression of ADRβ1. **Figure S5.** Ex vivo β-adrenergic stimulation elicits enteric glia calcium signaling. **Figure S6.** JellyOP mice with a mixed genetic background develop regular POI.

**Additional file 3: Video SA.** Isoprenaline. **Video SB.** Substance P.

## Data Availability

The datasets used and analyzed during the current study have been submitted to the GEO database under the accession number *GSE198889* with the following token: cdupiqaotjsnvgf.

## Abbreviations

EGC: Enteric glial cell
POI: Postoperative ileus
ENS: Enteric nervous system
CNS: Central nervous system
SNS: Sympathetic nervous system
GI: Gastro-intestinal
LPS: Lipopolysacharrid
Lap: Laparotomy
STX: Sympathetectomy (chemical)
NE: Norepinephrine
ME: Muscularis externa
AR: Adrenergic receptor
GO: Gene ontology
IM: Intestinal manipulation
6-OHDA: 6-Hydroxydopamine
GFAP: Glial fibrilliary acidic protein
SOX10: SRY-Box transcription factor 10
IL-6: Interleukin-6
IL-1β: Interleukin-1β
CCL2: C–C motif chemokine ligand 2
TNFα: Tumor necrosis factor α
IFNγ: Interferone γ
GDNF: Glial-cell derived neurotrophic factor
PLP1: Proteolipid protein 1
