# Supplementary material for: β-adrenergic signaling triggers enteric glial reactivity and acute enteric gliosis during surgery
Source: J Neuroinflammation. 2023 Nov 8;20:255. doi: 10.1186/s12974-023-02937-0 (PMC10631040; doi:10.1186/s12974-023-02937-0)
Supplement: Supplementary file 1 — Additional file 1: Method S1. RiboTag approach in the muscularis externa. Method S2. Construction of the JellyOP targeting vector for the mouse Rosa26 locus. Method S3. JellyOP animal creation. Method S4. Recombinant adeno-associated virus (rAAV) preparation. Table S1. Buffer and media. Table S2. Antibodies. Table S3. PCR primer. Table S4. Acute enteric gliosis GO-term. [file 12974_2023_2937_MOESM1_ESM.docx]

_______________________________________________________________

**Additional file 1**

Method S1: *RiboTag* approach in the *muscularis externa*

RiboTag IP was performed according to Leven et al. 2021. In brief: The small bowel muscle layer (*muscularis externa*; *ME*) was separated from the mucosal layer in ice-cold Krebs‑Henseleit buffer (Table S2). Due to the nature of the intestine, a swift separation and constant low temperature are integral to optimize the amount of RNA and its quality. As an additional precaution to protect specific mRNA, *ME* was immediately placed in RNAlater [Thermo Fisher Scientific, Waltham, MA, USA; AM7020]. Subsequently, about 100 mg of *ME* per mouse was lysed with a Precellys homogenizer [Bertin Instruments, Montigny‑le‑Bretonneux, FR] (3x 5000 rpm, 45 s; 5 min intermediate incubation on ice) in pre-cooled homogenization buffer (1 ml; Table S2). Lysate was centrifuged (10 min, 10000 g, 4°C), supernatants removed and separated into an “input control” portion (50 µl) and sample designated for immunoprecipitation (IP; ~950 µl). IP Samples were incubated with anti-HA antibody (5 μl; 1 mg/ml; Table S1) for 4 h at 4°C, 7 rpm on a sampler rotation device. Following the antibody binding, lysates were incubated overnight (4°C, 7 rpm) with 200 μl homogenization buffer equilibrated A/G dynabeads [Thermo Fisher Scientific, Waltham, MA, USA; 88802]. Beads with bound ribosomes were magnetically separated from unbound lysates (4°C, 1min) and washed off with high salt buffer (5 min three times, 4°C, 7 rpm; Table S2). Bead-Ribosome aggregates were subjected to a Qiagen micro kit [Qiagen, Hilden, NRW, DE; 74004] to elute glial-specific mRNA.

Method S2: Construction of the *JellyOP* targeting vector for the mouse Rosa26 locus

The cDNA for JellyOP was subcloned in pR26 CAG/GFP Asc, which was a generous gift of Dr. Ralf Kuehn [Addgene plasmid # 74285] (30) by DNA fragment assembly method using the in-vitro homologous recombination reaction (GeneArt® Seamless Cloning Kit [InvitrogenTM Life TechnologiesTM, ThermoFisher Scientific, Carlsbad, CA, USA]). In detail, the vector DNA pR26 CAG/GFP Asc was linearized by the endonuclease restriction enzymes AscI and subsequently treated by the large fragment of DNA Polymerase I (Klenow Fragment) [ThermoFisher Scientific, Waltham, MA, USA] to blunt the ends. Then the linearized DNA was dephosphorylated by FastAP thermosensitive alkaline phosphatase [ThermoFisher Scientific, Waltham, MA, USA] and later purified by QIAquick PCR Purification Kit [QIAGEN, Hilden, Germany]. The cDNA for JellyOP (31), was synthetized as strings DNA fragments [Invitrogen™ GeneArt™ Strings™, ThermoFisher Scientific, Regensburg, Germany]. Following the manufacturer’s specification, DNA fragment of the insert cDNA for JellyOP and the linearized DNA vector pR26 CAG/GFP Asc was assembled, which generated the construct named pR26 CAG/JellyOP/GFP. The resulted construct was further verified by sanger sequencing [Eurofins Genomics, Erbersberg, Germany]. Finally, to prepare this targeting vector DNA for microinjection, the verified plasmid DNA was obtained and extracted from the large bacteria culture using EndoFree Plasmid Maxi Kit [QIAGEN, Hilden, Germany].

Method S3: *JellyOP* animal creation

Gt(ROSA)26Sorem1(CAG-JellyOp-eGFP) mice were generated by CRISPR/Cas9 mediated gene-editing in zygotes according to (30). Briefly, Rosa26-crRNA and tracrRNA [IDT, Leuven, Belgium] were combined in TE buffer (#11-05-01-15, IDT) to a final concentration of 10 µM each and annealed (95°C, 5 min; cool down to room temperature with -0.2°C/sec; 22°C, 10 min). Injection mixes were assembled in TE microinjection buffer containing 50 ng/µl Cas9 protein (IDT), 0.6 µM annealed cr/tracr RNA, and 20 ng/µl pR26 CAG/JellyOP/GFP plasmid DNA. For pronuclear injection, zygotes were obtained by 1:1 mating of superovulated B6D2F1 females with B6D2F1 males [Charles River, Sulzfeld, Germany]. Pronuclear injection was performed according to standard procedures. Recovered embryos were cultured overnight at 37°C, 5% CO2 in G-TL medium [Vitrolife, Göteborg, Sweden] and transferred into the oviduct of pseudopregnant CD1 foster mice at the 2-cell stage. Offspring were genotyped and gene-edited alleles were separated by backcrossing of founder animals with CD1 mice. For optogenetic experiments generated *JellyOP* mice were crossbred with B6N.129-*Sox10^iCreERT2^/Rpl22^HA/+^/Ai14^fl/fl^* mice to enable activation of the LoxP site with tamoxifen in enteric glia cells.

Method S4: Recombinant adeno-associated virus (rAAV) preparation

rAAV2/1-GFAP-NLS-Cre-WPRE was prepared as previously described [1]. In brief, after transfection of HEK293T cells with pAAV-GFAP-NLS-Cre-WPRE and the helper plasmids pFΔ6, pRV1, and pH21, cells were lysed in 0.5% sodium deoxycholate (Sigma-Aldrich) and 50 units/ml Benzonase endonuclease (Millipore). HiTrapTM heparin columns (GE Healthcare) were used to purify AAV viral particles and the virus was concentrated to a final volume of 400 µl using Amicon Ultra Centrifugal Filters (Millipore). The purity of the virus was validated by SDS-PAGE followed by Coomassie Blue staining. Virus titer was determined by quantitative PCR (qPCR) with primers binding to the WPRE element.

**Table S1. Buffer and media.**

| **Buffer** | **Components** | |
| --- | --- | --- |
|  | NaCl 120 mM | Glucose 17.5 mM |
|  | KCl 5.9 mM | 2(H_2_O)CaCl_2_ 2.5 mM |
| Krebs-Henseleit | NaHCO_3_ 15.5 mM | 6(H_2_O)MgCl_2_ 1.17 mM |
|  | NaH_2_PO_4_ 1.4 mM |  |
|  |  |  |
|  |  |  |
|  | NaCl 126 mM | 2(H_2_O)CaCl_2_ 2.5 mM |
|  | KCl 2.5 mM | 6(H_2_O)MgCl_2_ 1.2 mM |
| Krebs-Henseleit (cell culture) | NaHCO_3_ 25 mM | Penicillin 100 IU/ml |
|  | NaH_2_PO_4_ 1.2 mM | Streptomycin 100 IU/ml |
|  |  | Amphotericin 2.5 µg/ml |
|  |  |  |
|  |  |  |
|  | DMEM |  |
| Dissociation buffer | Protease Type 1 0.25 mg/ml |  |
|  | Collagenase A 1 mg/ml |  |
|  |  |  |
|  |  |  |
|  | Neurobasal medium | Penicillin 100 IU/ml |
| Proliferation media | FGF 20 ng/ml | Streptomycin 100 IU/ml |
|  | EGF 20 ng/ml | Amphotericin 2.5 µg/ml |
|  |  |  |
|  |  |  |
|  | Neurobasal medium | Penicillin 100 IU/ml |
| Differentiation media | B27 | Streptomycin 100 IU/ml |
|  | N2 | Amphotericin 2.5 µg/ml |
|  | EGF 2 ng/ml |  |
|  |  |  |
|  | 50 mM Tris/HCl | 100 mM KCl |
|  | 1% NP-40 | 12 mM MgCl_2_ |
| Homogenization (lysis) | 1 mg/ml Heparin | 1 mM DTT |
|  | 100 µg/ml Cycloheximide | 1x Protease Inhibitor P8340 |
|  | 200 u/ml RNAsin |  |
|  |  |  |
|  |  |  |
| High salt buffer (washing) | 50 mM Tris/HCl | 300 mM KCl |
|  | 1% NP-40 | 12 mM MgCl_2_ |
|  | 100 µg/ml Cycloheximide | 0.5 mM DTT |
|  |  |  |

**Table S2. Antibodies.**

| **Host** | **Target** | **Fluorophor** | **Clone** | **Supplier** | **Art. No.** | **Use** |
| --- | --- | --- | --- | --- | --- | --- |
| Goat | Sox10 | --- | Polyclonal | SantaCruz Biotech | *discontinued* | IHC |
| Goat | Sox10 | --- | Polyclonal | Self-made | --- | IHC |
| Rabbit | Ki67 | --- | SP6 | Abcam | Ab16667 | IHC |
| Rabbit | HA | --- | C29F4 | Cell signaling | 3724S | IHC |
| Rabbit | TH | --- | Polyclonal | Sigma-Aldrich | AB152 | IHC |
| Rabbit | βAR | --- | Polyclonal | Cusabio | PA000937 | IHC |
| Rabbit | α2aAR | --- | Polyclonal | Alomone Labs | AAR-020 | ICC |
| Rabbit | FOS | --- | Polyclonal | SantaCruz | *discontinued* | IHC |
| Rabbit | IgG | --- | Polyclonal | Dianova | 011-000-003 | IHC |
| Rabbit | GFP | --- | Polyclonal | SYSY | 132002 | IHC |
| Mouse | Tubb3 | --- | Tuj1 | Biolegend | 801201 | IHC |
| Mouse | GFAP | Alexa594 | 2E1.E9 | Biolegend | 644708 | IHC |
| Chicken | Map2 | --- | Polyclonal | Biolegend | BLD-822501 | IHC |
| Donkey | Rabbit | FITC | Polyclonal | Dianova | 711-095-152 | IHC/ICC |
| Donkey | Rabbit | Alexa647 | Polyclonal | Dianova | 711-606-152 | IHC/ICC |
| Donkey | Goat | Alexa488 | Polyclonal | Thermo Scientific | A-11055 | IHC |
| Donkey | Goat | CF647 | Polyclonal | Sigma-Aldrich | SAB4600175 | IHC |
| Donkey | Chicken | FITC | Polyclonal | Biozol | 703-095-155 | IHC |
| Donkey | Chicken | CF633 | Polyclonal | Sigma-Aldrich | SAB4600127 | IHC |
| Donkey | Mouse | Alexa488 | Polyclonal | Dianova | 715-545-151 | IHC |
| Donkey | Mouse | CF647 | Polyclonal | Sigma-Aldrich | SAB4600176 | IHC |
| Mouse | HA | --- | 16-B-12 | Biolegend | 901514 | IP |
| Chicken | Vimentin | --- | Poly29191 | Biolegend | BLD-919101 | WB |
| Rabbit | GFAP | --- | Polyclonal | SySy | 173002 | WB |
| Rabbit | pPKA | --- | 100G7E | Cell signaling | 9624 | WB |
| Mouse | β-actin | --- | AC-74 | Sigma-Aldrich | A5316 | WB |
| Donkey | Mouse | DyLight 800 | Polyclonal | Thermo Scientific | SA5-10172 | WB |
| Donkey | Chicken | IRDye 680 | Polyclonal | Li-Cor | 926-68075 | WB |
| Donkey | Rabbit | IRDye 680 | Polyclonal | Li-Cor | 926-68073 | WB |

**Table S3. PCR primer.**

| **Gene** | **Forward primer** | **Reverse primer** |
| --- | --- | --- |
| *18S* | GTAACCCGTTGAACCCCATT | CCATCCAATCGGTAGTAGCG |
| *Pgk1* | Assay: QT00306558 | |
| *GAPDH* | GGGAAGCCCATCACCATCTT | GCCTCACCCCATTTGATGTT |
| *βActin* | AGAGGGAAATCGTGCGTGAC | CAATAGTGATGACCTGGCCGT |
| *Tubb4* | Assay: QT00251664 | |
| *Il6* | AAGTCGGAGGCTTAATTACACATGT | CCATTGCACAACTCTTTTCTCATT |
| *Ccl2* | AGGTCCCTGTCATGCTTCTG | TCTGGACCCATTCCTTCTTG |
| *FosB* | ATGGGCTCTCCTGTCAACAC | ACGGAGGAGACCAGAGTGG |
| *Stat3* | AGCTGGACACACGCTACCT | AGGAATCGGCTATATTGCTGGT |
| *Gfap* | ACATCGAGATCGCCACCTAC | CCTTCTGACACGGATTTGGT |
| *Nestin* | AGATCGCTCAGATCCTGGAA | AGGTGTCTGCAAGCGAGAGT |
| *Adra1a* | TTGAAATTCGGGAAGAAGGA | GAGAAGAAAGCCGCCAAGAC |
| *Adra1b* | AGGCAGCTGTTGAAGTAGCC | TCTTCATCGCTCTCCCACTT |
| *Adra1d* | ATTGAAGTAGCCCAGCCAGA | GCTGGTTCCCCTTTTTCTTC |
| *Adra2a* | TCTGGTCGTTGATCTTGCAG | CATCTCGGCTGTCATCTCCT |
| *Adra2b* | CAAAGCCTTTCATCTCCCTG | CTGGCCTCGACCTCACTAAG |
| *Adra2c* | CCACCTCACTCGGTTACAGG | CTACAAGCGCACTCTCCAATC |
| *Adrb1* | AAGTCCAGAGCTCGCAGAAG | GCTGATCTGGTCATGGGATT |
| *Adrb2* | TAGCGATCCACTGCAATCAC | ATTTTGGCAACTTCTGGTGC |
| *Adrb3* | GGGGAAGGTAGAAGGAGACG | ACAGGAATGCCACTCCAATC |

| **Table S4. Acute enteric gliosis GO-term.** | | | | |
| --- | --- | --- | --- | --- |
| **Gene** | **Fold** | **Mean Counts (IM3h)** | **Mean Counts (Naive)** | **Source** |
| *Tnfaip3* | 20,25 | 27,00 | 1,33 | [6] |
| *Ell2* | 20,17 | 524,53 | 26,01 | Fold > ±10 |
| *Wnk3* | 19,83 | 59,50 | 3,00 | Fold > ±10 |
| *Sphk1* | 19,78 | 609,50 | 30,82 | [7, 10, 11] |
| *Phlda1* | 19,10 | 853,25 | 44,67 | Fold > ±10 |
| *Maff* | 18,96 | 455,00 | 24,00 | Fold > ±10 |
| *Trib1* | 18,78 | 3574,50 | 190,33 | Fold > ±10 |
| *Sdc4* | 18,36 | 14036,75 | 764,33 | Fold > ±10 |
| *Runx1* | 18,23 | 905,25 | 49,67 | Fold > ±10 |
| *Rgs16* | 17,41 | 748,50 | 43,00 | Fold > ±10 |
| *Snhg4* | 17,09 | 451,35 | 26,42 | Fold > ±10 |
| *Atp8b1* | 16,31 | 951,50 | 58,33 | Fold > ±10 |
| *Bach1* | 16,13 | 2118,25 | 131,33 | Fold > ±10 |
| *Utp4* | 16,08 | 831,00 | 51,67 | Fold > ±10 |
| *Dusp4* | 16,03 | 197,75 | 12,33 | Fold > ±10 |
| *Gadd45b* | 15,92 | 663,25 | 41,67 | [7] |
| *Pfkfb3* | 15,05 | 918,00 | 61,00 | Fold > ±10 |
| *Uck2* | 14,91 | 1535,75 | 103,00 | Fold > ±10 |
| *Nr4a1* | 14,86 | 1396,99 | 94,00 | Fold > ±10 |
| *Syt6* | 14,73 | 1360,00 | 92,33 | Fold > ±10 |
| *Lingo1* | 14,43 | 158,75 | 11,00 | [3] |
| *Bcat1* | 14,25 | 194,75 | 13,67 | Fold > ±10 |
| *Chst11* | 14,10 | 437,25 | 31,00 | Fold > ±10 |
| *Zfp469* | 14,05 | 960,00 | 68,33 | Fold > ±10 |
| *Rrp8* | 13,83 | 474,75 | 34,33 | Fold > ±10 |
| *Ppp1r15a* | 13,21 | 1677,50 | 127,00 | Fold > ±10 |
| *Zfp597* | 13,20 | 162,75 | 12,33 | Fold > ±10 |
| *Taf4b* | 13,11 | 236,00 | 18,00 | Fold > ±10 |
| *Gem* | 13,01 | 1045,25 | 80,33 | Fold > ±10 |
| *Fndc4* | 12,95 | 112,25 | 8,67 | Fold > ±10 |
| *Cd44* | 12,86 | 3766,43 | 292,80 | [7, 8, 11] |
| *Spred2* | 12,72 | 852,25 | 67,00 | Fold > ±10 |
| *Tuba1c* | 12,60 | 2418,14 | 191,96 | [2] |
| *Nr4a2* | 12,55 | 631,50 | 50,33 | [4] |
| *Hilpda* | 12,43 | 281,75 | 22,67 | Fold > ±10 |
| *Cldn5* | 12,42 | 149,00 | 12,00 | Fold > ±10 |
| *Dbpht2* | 12,30 | 47,75 | 3,88 | Fold > ±10 |
| *Mpzl2* | 12,28 | 246,04 | 20,03 | Fold > ±10 |
| *Grwd1* | 12,21 | 350,00 | 28,67 | Fold > ±10 |
| *Frmd4a* | 12,20 | 5600,38 | 459,05 | Fold > ±10 |
| *Zfp36* | 12,08 | 1006,98 | 83,33 | Fold > ±10 |
| *Ddx21* | 11,82 | 5920,25 | 501,00 | Fold > ±10 |
| *Elfn1* | 11,79 | 55,00 | 4,67 | Fold > ±10 |
|  |  |  |  |  |
| **Gene** | **Fold** | **Mean Counts (IM3h)** | **Mean Counts (Naive)** | **Source** |
| *Mak16* | 11,79 | 1005,40 | 85,31 | Fold > ±10 |
| *Utp18* | 11,78 | 227,75 | 19,33 | Fold > ±10 |
| *Azin1* | 11,75 | 6715,06 | 571,33 | Fold > ±10 |
| *Map3k6* | 11,60 | 116,00 | 10,00 | Fold > ±10 |
| *Gbp2* | 11,57 | 2313,25 | 200,00 | [7, 11] |
| *Has1* | 11,39 | 421,25 | 37,00 | Fold > ±10 |
| *Map2k3* | 11,24 | 2184,52 | 194,33 | Fold > ±10 |
| *Eif1a* | 11,21 | 1210,24 | 108,00 | Fold > ±10 |
| *Emp1* | 11,19 | 2845,84 | 254,33 | [7, 10, 11] |
| *Coq10b* | 11,13 | 397,13 | 35,67 | Fold > ±10 |
| *Yrdc* | 11,13 | 159,50 | 14,33 | Fold > ±10 |
| *Dusp14* | 11,08 | 218,00 | 19,67 | Fold > ±10 |
| *Snhg6* | 10,88 | 36,25 | 3,33 | Fold > ±10 |
| *Capn12* | 10,81 | 1357,00 | 125,59 | Fold > ±10 |
| *Sertad1* | 10,68 | 1014,50 | 95,00 | Fold > ±10 |
| *Sox4* | 10,53 | 13551,00 | 1287,08 | Fold > ±10 |
| *Socs3* | 10,51 | 518,50 | 49,33 | [4, 6] |
| *Pprc1* | 10,48 | 412,25 | 39,33 | Fold > ±10 |
| *Fem1b* | 10,41 | 3049,75 | 293,00 | Fold > ±10 |
| *Spa17* | 10,29 | 46,32 | 4,50 | Fold > ±10 |
| *Sept9* | 10,27 | 554,75 | 54,00 | Fold > ±10 |
| *Tmed10-ps* | 10,25 | 287,62 | 28,05 | Fold > ±10 |
| *Ifi211* | 10,24 | 176,62 | 17,25 | Fold > ±10 |
| *Cemip2* | 10,11 | 1857,75 | 183,67 | Fold > ±10 |
| *Tubb6* | 10,08 | 1875,79 | 186,17 | [7] |
| *Mbip* | 10,03 | 26,75 | 2,67 | Fold > ±10 |
| *Jun* | 9,73 | 6009,75 | 617,67 | [4, 6] |
| *Sox9* | 9,44 | 330,50 | 35,00 | [4, 9] |
| *Vgf* | 9,33 | 1378,25 | 147,67 | [7] |
| *Nfkbie* | 9,16 | 58,00 | 6,33 | [6] |
| *Tnc* | 8,96 | 3059,79 | 341,67 | [12] |
| *Timp1* | 8,95 | 435,75 | 48,67 | [4, 7, 11] |
| *Thbs1* | 8,82 | 2449,55 | 277,67 | [2, 4] |
| *Flnc* | 8,60 | 3744,32 | 435,33 | [7] |
| *Cebpb* | 8,33 | 569,25 | 68,33 | [6] |
| *Slc39a14* | 8,12 | 208,50 | 25,67 | [7] |
| *Cxcl10* | 8,04 | 152,75 | 19,00 | [4, 5, 7, 11] |
| *Ptgs2* | 7,43 | 215,50 | 29,00 | [7, 10, 11] |
| *Gadd45g* | 6,87 | 618,25 | 90,00 | [6] |
| *Fkbp5* | 6,75 | 441,11 | 65,40 | [7, 10, 11] |
| *Bcl6* | 6,50 | 750,00 | 115,33 | [8] |
| *Sbno2* | 6,42 | 2499,50 | 389,33 | [6, 7] |
| *Akap12* | 6,29 | 12380,14 | 1968,33 | [7] |
|  |  |  |  |  |
|  |  |  |  |  |
|  |  |  |  |  |
| **Gene** | **Fold** | **Mean Counts (IM3h)** | **Mean Counts (Naive)** | **Source** |
| *Gfap* | 5,99 | 4748,75 | 792,33 | [3, 4, 7, 8, 11] |
| *Fas* | 5,95 | 168,50 | 28,33 | [6] |
| *Slc10a6* | 5,79 | 229,75 | 39,67 | [7, 11] |
| *Gbp5* | 5,74 | 376,75 | 65,67 | [6] |
| *Col28a1* | 5,70 | 117,75 | 20,67 | [6] |
| *Fgf2* | 5,48 | 731,97 | 133,53 | [4] |
| *Tspan4* | 5,42 | 219,19 | 40,44 | [7] |
| *Casp4* | 5,12 | 140,00 | 27,33 | [6] |
| *Nfkbiz* | 4,83 | 676,50 | 140,00 | [6] |
| *Cryab* | 4,58 | 3731,67 | 814,00 | [4, 8] |
| *S100a10* | 4,58 | 2538,00 | 554,33 | [7, 10, 11] |
| *Vegfa* | 4,54 | 466,00 | 102,66 | [4] |
| *Gcnt2* | 4,09 | 1258,49 | 307,33 | [7] |
| *Ier3* | 4,01 | 918,50 | 229,00 | [7] |
| *Ggta1* | 3,91 | 572,75 | 146,33 | [7, 10, 11] |
| *Smad3* | 3,90 | 489,75 | 125,67 | [4] |
| *Osmr* | 3,83 | 270,50 | 70,67 | [11] |
| *Tnfaip2* | 3,75 | 316,50 | 84,33 | [6] |
| *Ccl7* | 3,70 | 37,00 | 10,00 | [4] |
| *Anxa2* | 3,59 | 4279,24 | 1191,67 | [7] |
| *Icam1* | 3,55 | 316,49 | 89,14 | [7] |
| *Gbp3* | 3,48 | 776,93 | 222,95 | [7] |
| *Irf1* | 3,35 | 1437,36 | 429,33 | [4, 6] |
| *Bcl3* | 3,30 | 231,75 | 70,33 | [6] |
| *Nfe2l2* | 3,22 | 901,00 | 279,67 | [4] |
| *Stat2* | 2,95 | 257,50 | 87,33 | [4] |
| *Myd88* | 2,88 | 236,25 | 82,00 | [4] |
| *Lgals3* | 2,78 | 217,75 | 78,33 | [7] |
| *Slc11a2* | 2,74 | 329,75 | 120,33 | [6] |
| *Crispld2* | 2,73 | 1847,75 | 675,67 | [6, 7] |
| *Klf5* | 2,71 | 370,75 | 136,67 | [7] |
| *Gsr* | 2,70 | 134,25 | 49,67 | [7] |
| *Fcgr2b* | 2,70 | 107,00 | 39,67 | [6] |
| *Tm4sf1* | 2,51 | 518,00 | 206,33 | [7, 10, 11] |
| *Stat3* | 2,50 | 4910,97 | 1966,67 | [4] |
| *Glul* | 2,43 | 1306,75 | 538,33 | [3] |
| *Ctnnb1* | 2,36 | 318,95 | 134,95 | [9] |
| *Sema4a* | 2,23 | 144,25 | 64,67 | [4] |
| *Tgfb1* | 2,19 | 108,75 | 49,67 | [4] |
| *S1pr3* | 2,19 | 1187,25 | 543,00 | [11] |
| *Gap43* | 2,12 | 1580,00 | 747,00 | [7] |
| *Nek6* | 2,04 | 239,43 | 117,16 | [7] |
| *Pdlim4* | 1,85 | 818,75 | 443,00 | [6] |
|  |  |  |  |  |
|  |  |  |  |  |
|  |  |  |  |  |
| **Gene** | **Fold** | **Mean Counts (IM3h)** | **Mean Counts (Naive)** | **Source** |
| *Csf1* | 1,85 | 449,50 | 243,33 | [6] |
| *Vim* | 1,83 | 9233,05 | 5043,28 | [4, 7, 11] |
| *Hspb1* | 1,68 | 3239,27 | 1933,24 | [11] |
| *Cpe* | 1,64 | 4351,25 | 2650,67 | [3] |
| *Zfp36l1* | 1,58 | 2221,25 | 1404,33 | [3] |
| *H2-D1* | 1,58 | 4755,20 | 3016,37 | [7, 10, 11] |
| *Sorbs1* | -1,57 | 3670,74 | 5769,99 | [7] |
| *Gja1* | -1,62 | 538,75 | 871,33 | [3] |
| *Ccn2* | -1,68 | 701,41 | 1175,62 | [12] |
| *Serping1* | -1,85 | 240,75 | 445,67 | [7, 10, 11] |
| *Prkaca* | -1,94 | 317,00 | 614,67 | [4] |
| *Mrvi1* | -2,08 | 568,75 | 1181,34 | [2] |
| *Klhl30* | -2,23 | 54,75 | 122,00 | [2] |
| *Sparcl1* | -2,39 | 3767,54 | 8985,82 | [12] |
| *Myom1* | -2,51 | 1210,75 | 3036,01 | [2] |
| *Dcn* | -2,55 | 667,75 | 1704,33 | [6] |
| *Kcnj10* | -2,56 | 114,50 | 293,33 | [8] |
| *Hspb6* | -2,63 | 523,50 | 1378,99 | [7] |
| *Des* | -2,75 | 2369,25 | 6517,00 | [2] |
| *Myl9* | -2,76 | 5544,25 | 15281,67 | [2] |
| *Aoc3* | -2,85 | 106,25 | 303,00 | [2] |
| *Rassf10* | -3,63 | 23,50 | 85,33 | [6] |
| *Pdlim3* | -3,73 | 865,50 | 3229,16 | [2] |
| *Pln* | -3,77 | 68,15 | 256,62 | [2] |
| *C3* | -5,43 | 616,75 | 3352,00 | [13] |
| *Lypd8* | -12,00 | 11,00 | 132,00 | Fold > ±10 |
| *Vmn2r3* | -12,09 | 2,00 | 24,18 | [6] |
| *mt-Ti* | -14,15 | 3,25 | 46,00 | Fold > ±10 |
| *mt-Ty* | -14,29 | 5,25 | 75,00 | Fold > ±10 |
| *Tatdn1* | -16,51 | 478,95 | 7909,57 | Fold > ±10 |
| *Elp6* | -17,00 | 175,12 | 2977,12 | Fold > ±10 |
| *Lrrc17* | -17,33 | 233,21 | 4040,47 | Fold > ±10 |
| *Akap5* | -19,37 | 251,55 | 4871,44 | Fold > ±10 |
| *Nmrk2* | -19,62 | 7,50 | 147,17 | Fold > ±10 |
| *Lrrn4* | -24,39 | 12,75 | 311,00 | Fold > ±10 |
| *Rspo1* | -29,33 | 2,50 | 73,33 | Fold > ±10 |
| *A3galt2* |  | 29,50 | 0,00 | × |
| *Acod1* |  | 34,25 | 0,00 | × |
| *Amigo3* |  | 18,75 | 0,00 | × |
| *Areg* |  | 187,75 | 0,00 | × |
| *Asgr1* |  | 26,25 | 0,00 | × |
| *Ccdc7a* |  | 95,75 | 0,00 | × |
| *Ciart* |  | 58,75 | 0,00 | × |
|  |  |  |  |  |
|  |  |  |  |  |
|  |  |  |  |  |
| **Gene** | **Fold** | **Mean Counts (IM3h)** | **Mean Counts (Naive)** | **Source** |
| *Col24a1* |  | 17,75 | 0,00 | × |
| *Dusp2* |  | 26,50 | 0,00 | × |
| *Efcab5* |  | 19,75 | 0,00 | × |
| *Epha6* |  | 16,00 | 0,00 | × |
| *Fam71a* |  | 136,25 | 0,00 | × |
| *Fgf18* |  | 20,75 | 0,00 | × |
| *Fosl1* |  | 708,58 | 0,00 | × |
| *Gpr3* |  | 36,00 | 0,00 | × |
| *Il18rap* |  | 35,75 | 0,00 | × |
| *Il1r2* |  | 18,75 | 0,00 | × |
| *Klhl40* |  | 54,75 | 0,00 | × |
| *Klk9* |  | 214,75 | 0,00 | × |
| *March3* |  | 16,00 | 0,00 | × |
| *Mctp2* |  | 22,00 | 0,00 | × |
| *Olfr267* |  | 18,25 | 0,00 | × |
| *Olfr458* |  | 20,75 | 0,00 | × |
| *Olfr597* |  | 15,50 | 0,00 | × |
| *Prss35* |  | 21,75 | 0,00 | × |
| *Ptx3* |  | 140,00 | 0,00 | × |
| *Slfn4* |  | 32,25 | 0,00 | × |
| *Sstr2* |  | 52,75 | 0,00 | × |
| *Tas2r143* |  | 22,50 | 0,00 | × |
| *Tecta* |  | 27,75 | 0,00 | × |
| *Tgm1* |  | 33,25 | 0,00 | × |
| *Tnni2* |  | 16,25 | 0,00 | × |
| *Trim30c* |  | 16,54 | 0,00 | × |
| *Trim69* |  | 20,25 | 0,00 | × |
| *Ucn2* |  | 236,00 | 0,00 | × |
| *Zfp711* |  | 21,50 | 0,00 | × |
| *Il6* |  | 39,25 | 0,00 | ×[4, 5] |

| **Gene** | **Fold** | **Mean Counts (IM3h)** | **Mean Counts (Naive)** | **Source** |
| --- | --- | --- | --- | --- |
| *Egr2* | 665,25 | 221,75 | 0,33 | [2] |
| *Serpine1* | 187,12 | 4303,86 | 23,00 | Fold > ±10 |
| *Mt2* | 173,28 | 36811,61 | 212,44 | [3] |
| *Ccl2* | 135,33 | 1353,25 | 10,00 | [4–6] |
| *Hbegf* | 115,57 | 20070,66 | 173,67 | Fold > ±10 |
| *Egr1* | 87,29 | 12075,58 | 138,33 | [2] |
| *Gdnf* | 78,39 | 4755,75 | 60,67 | [4] |
| *Rn7sk* | 65,74 | 316,61 | 4,82 | Fold > ±10 |
| *Dusp5* | 64,94 | 2684,00 | 41,33 | Fold > ±10 |
| *Ccn1* | 55,86 | 2234,50 | 40,00 | Fold > ±10 |
| *Atf3* | 53,79 | 878,50 | 16,33 | Fold > ±10 |
| *Mt1* | 45,68 | 31064,47 | 680,00 | [3] |
| *Srxn1* | 45,31 | 8307,50 | 183,33 | [7] |
| *Fos* | 44,80 | 1269,25 | 28,33 | [4, 8] |
| *Adamts1* | 41,71 | 2641,50 | 63,33 | Fold > ±10 |
| *Cxcl1* | 38,19 | 152,75 | 4,00 | [4, 5, 7] |
| *Rpsa-ps12* | 37,61 | 917,40 | 24,40 | Fold > ±10 |
| *Cntd1* | 36,73 | 73,17 | 1,99 | Fold > ±10 |
| *Csrnp1* | 34,12 | 830,25 | 24,33 | Fold > ±10 |
| *Junb* | 33,65 | 1532,96 | 45,56 | [6] |
| *Mmp3* | 31,13 | 41,50 | 1,33 | [4, 6] |
| *Il11* | 30,78 | 256,50 | 8,33 | [4] |
| *Errfi1* | 30,13 | 853,75 | 28,33 | Fold > ±10 |
| *Rcan1* | 29,64 | 4040,75 | 136,33 | Fold > ±10 |
| *Tnfaip6* | 29,56 | 384,25 | 13,00 | Fold > ±10 |
| *Btc* | 29,25 | 663,00 | 22,67 | Fold > ±10 |
| *Hmox1* | 28,67 | 669,00 | 23,33 | [7] |
| *Vasn* | 26,30 | 4049,50 | 154,00 | Fold > ±10 |
| *Cxcl5* | 26,18 | 96,00 | 3,67 | [6] |
| *Map3k8* | 25,06 | 200,50 | 8,00 | [6] |
| *Nes* | 23,78 | 8330,81 | 350,33 | [4, 7, 9] |
| *Kdm6b* | 22,04 | 6267,00 | 284,33 | Fold > ±10 |
| *Tnfrsf12a* | 21,82 | 2021,75 | 92,67 | [7] |
| *Cdt1* | 21,61 | 273,75 | 12,67 | Fold > ±10 |
| *Ifrd1* | 21,36 | 4422,53 | 207,00 | Fold > ±10 |
| *Lif* | 21,36 | 192,25 | 9,00 | [4] |
| *Ets1* | 20,68 | 1709,25 | 82,67 | Fold > ±10 |
| *Nfkbia* | 20,30 | 19848,25 | 977,67 | Fold > ±10 |
| *Hmga1* | 20,28 | 3542,45 | 174,69 | [7] |
| *Ier5l* | 20,27 | 1060,75 | 52,33 | Fold > ±10 |

× **Naive 0 counts; IM3h > 15 counts**

References

1. van Loo KMJ, Schaub C, Pernhorst K, Yaari Y, Beck H, Schoch S, Becker AJ. Transcriptional regulation of T-type calcium channel CaV3.2: bi-directionality by early growth response 1 (Egr1) and repressor element 1 (RE-1) protein-silencing transcription factor (REST). J Biol Chem. 2012;287:15489–501. doi:10.1074/jbc.M111.310763.

2. Orre M, Kamphuis W, Osborn LM, Jansen AHP, Kooijman L, Bossers K, Hol EM. Isolation of glia from Alzheimer's mice reveals inflammation and dysfunction. Neurobiol Aging. 2014;35:2746–60. doi:10.1016/j.neurobiolaging.2014.06.004.

3. Mathys H, Davila-Velderrain J, Peng Z, Gao F, Mohammadi S, Young JZ, et al. Single-cell transcriptomic analysis of Alzheimer's disease. Nature. 2019;570:332–7. doi:10.1038/s41586-019-1195-2.

4. Sofroniew MV. Astrogliosis. Cold Spring Harb Perspect Biol. 2014;7:a020420. doi:10.1101/cshperspect.a020420.

5. Nieves MD, Furmanski O, Doughty ML. Sensorimotor dysfunction in a mild mouse model of cortical contusion injury without significant neuronal loss is associated with increases in inflammatory proteins with innate but not adaptive immune functions. J Neurosci Res. 2021;99:1533–49. doi:10.1002/jnr.24766.

6. Rosenbaum C, Schick MA, Wollborn J, Heider A, Scholz C-J, Cecil A, et al. Activation of Myenteric Glia during Acute Inflammation In Vitro and In Vivo. PLoS One. 2016;11:e0151335. doi:10.1371/journal.pone.0151335.

7. Zamanian JL, Xu L, Foo LC, Nouri N, Zhou L, Giffard RG, Barres BA. Genomic analysis of reactive astrogliosis. Journal of Neuroscience. 2012;32:6391–410. doi:10.1523/JNEUROSCI.6221-11.2012.

8. Schirmer L, Velmeshev D, Holmqvist S, Kaufmann M, Werneburg S, Jung D, et al. Neuronal vulnerability and multilineage diversity in multiple sclerosis. Nature. 2019;573:75–82. doi:10.1038/s41586-019-1404-z.

9. Hara M, Kobayakawa K, Ohkawa Y, Kumamaru H, Yokota K, Saito T, et al. Interaction of reactive astrocytes with type I collagen induces astrocytic scar formation through the integrin-N-cadherin pathway after spinal cord injury. Nat Med. 2017;23:818–28. doi:10.1038/nm.4354.

10. Rakers C, Schleif M, Blank N, Matušková H, Ulas T, Händler K, et al. Stroke target identification guided by astrocyte transcriptome analysis. Glia. 2019;67:619–33. doi:10.1002/glia.23544.

11. Fujita A, Yamaguchi H, Yamasaki R, Cui Y, Matsuoka Y, Yamada K-I, Kira J-I. Connexin 30 deficiency attenuates A2 astrocyte responses and induces severe neurodegeneration in a 1-methyl-4-phenyl-1,2,3,6-tetrahydropyridine hydrochloride Parkinson's disease animal model. J Neuroinflammation. 2018;15:227. doi:10.1186/s12974-018-1251-0.

12. Jones EV, Bouvier DS. Astrocyte-secreted matricellular proteins in CNS remodelling during development and disease. Neural Plast. 2014;2014:321209. doi:10.1155/2014/321209.

13. Liddelow SA, Guttenplan KA, Clarke LE, Bennett FC, Bohlen CJ, Schirmer L, et al. Neurotoxic reactive astrocytes are induced by activated microglia. Nature. 2017;541:481–7. doi:10.1038/nature21029.
