# Supplementary material for: β-adrenergic signaling triggers enteric glial reactivity and acute enteric gliosis during surgery
Source: J Neuroinflammation. 2023 Nov 8;20:255. doi: 10.1186/s12974-023-02937-0 (PMC10631040; doi:10.1186/s12974-023-02937-0)
Supplement: Supplementary file 2 — Additional file 2: Figure S1. Characterization of POI in Sox10iCreERT2/Rpl22HA/+ mice. Figure S2. Sympathetic signaling is involved in enteric glia functions. Figure S3. Laparotomy affects molecular functions. Figure S4. Effect of NE and tissue expression of ADRβ1. Figure S5. Ex vivo β-adrenergic stimulation elicits enteric glia calcium signaling. Figure S6. JellyOP mice with a mixed genetic background develop regular POI. [file 12974_2023_2937_MOESM2_ESM.docx]

_______________________________________________________________


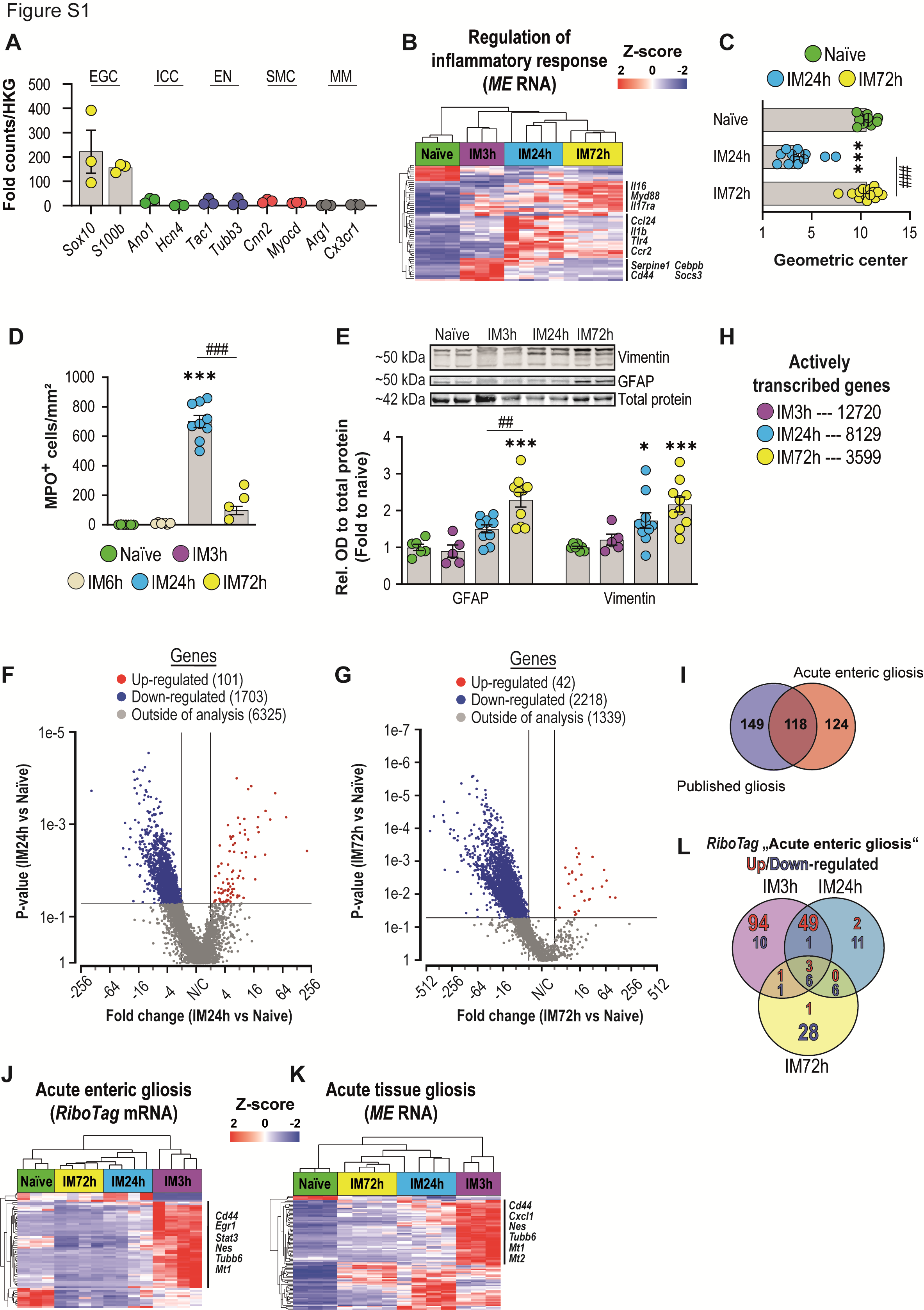


**Figure S1. Characterization of POI in *Sox10^iCreERT2^/Rpl22^HA/+^* mice.**

**(A)** Analysis for genes (mean ± SEM) of common cell types (enteric glial cells (EGC); interstitial cells of Cajal (ICC); Enteric neurons (EN); Smooth muscle cells (SMC); Muscularis macrophages (MM) in *Sox10^iCreERT2^/Rpl22^HA/+^ RiboTag* mRNA (Counts; *Actb/Hsp90ab1/Rplp1*, n = 3 mice per condition). **(B)** RNA‑Seq heat maps for naïve, IM3h, IM24h, and IM72h samples of *ME* RNA "regulation of inflammatory response" induction and indication of genes. (n = 3-4 mice per condition). **(C)**GI transit (mean ± SEM) for naïve, IM24h, and IM72h animals (n = 11-14 mice per condition; two-way ANOVA, * to naïve, # to IM24h, ***/### < 0.001). **(D)** Quantification of myeloidperoxidase^+^ (MPO) cells/mm^2^ (mean ± SEM; n = 4-9 mice per condition; two-way ANOVA, * to naïve, # to IM24h, ***/### < 0.001). **(E)**SDS‑PAGE and corresponding densitometry (mean ± SEM) of *Sox10^iCreERT2^/Rpl22^HA/+^* small bowel *ME* lysates stained for Vimentin (VIM) (~50 kDa) and GFAP (~50 kDa). BioRad stainless total protein band (42 kDa) was used as a loading control. (n = 5‑10 mice per condition; one‑way ANOVA, * to naive, # to IM24h, *** < 0.001, ## < 0.01, * < 0.05). **(F, G)** Volcano plots for actively transcribed genes at IM24h (F) and IM72h (G) in *Sox10^iCreERT2^/Rpl22^HA/+^* EGCs with DEGs (p‑value < 0.05, > ±2 fold) marked in red (upregulated) and blue (downregulated). **(H)**Actively transcribed genes at IM3h, IM24h, and IM72h. **(I)** Venn diagram of genes overlapping between published gliosis genes and the “acute enteric gliosis” GO-term at IM3h. **(J, K)** RNA‑Seq heat maps for *Sox10^iCreERT2^/Rpl22^HA/+^ RiboTag* mRNA (J) and *ME* RNA (K) filtered for the GO-term ”acute enteric gliosis” at different time points and implication of POI hallmark genes. (n = 3‑4 mice per condition) **(L)** Venn diagram of differentially regulated genes (DEG) in the “acute enteric gliosis” GO-term across different time points for *Sox10^iCreERT2^/Rpl22-HA* *RiboTag* mRNA samples.


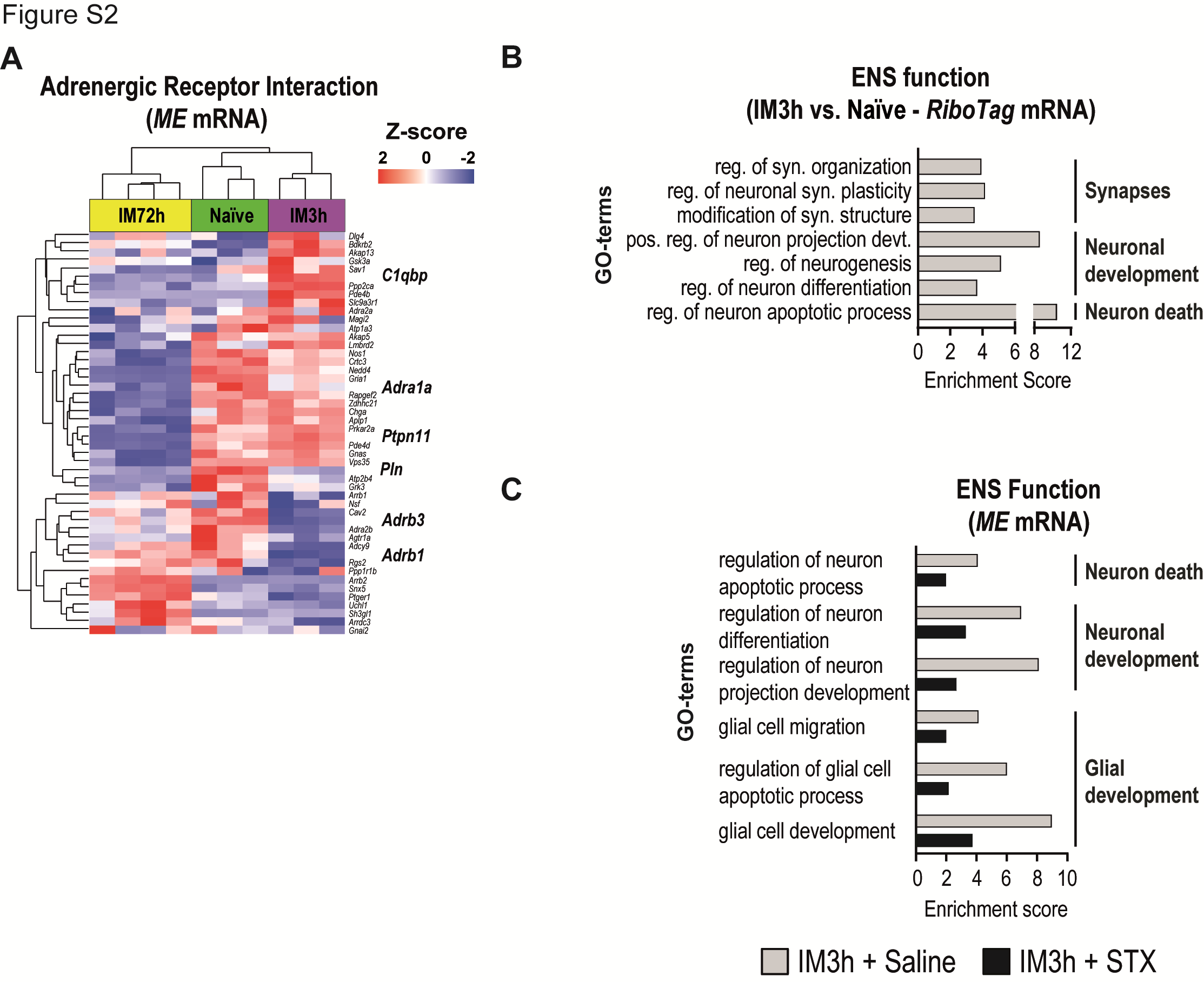


**Figure S2. Sympathetic signaling is involved in enteric glia functions.**

**(A)** Heat map for the novel GO-term “adrenergic receptor interaction” in naïve, IM3h, and IM72h samples of *ME* RNA and highlight on selected genes influencing signal transduction. (n = 3-4 mice per condition) **(B)** Analysis of enriched GO-terms in mRNA from *Sox10^iCreERT2^/Rpl22^HA/+^* EGCs related to ENS activity. **(C)**Analysis of enriched GO-terms in IM3h *ME* RNA and comparatively reduced in IM3h/STX samples related to ENS function.


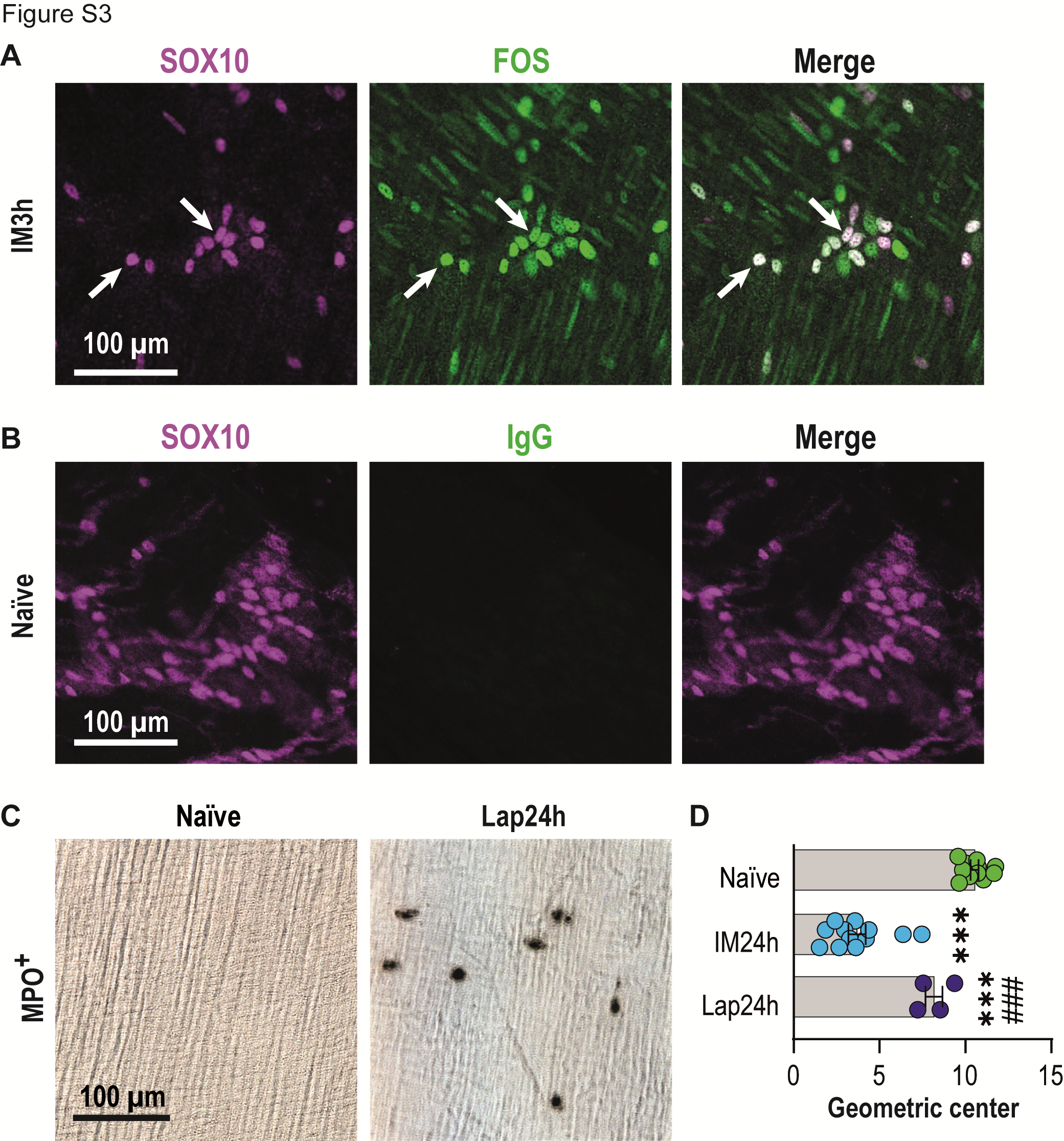


**Figure S3. Laparotomy affects molecular functions.**

**(A)** Confocal images of immunohistological stainings of SOX10 (magenta) and FOS (green) expression in whole mounts of IM3h small bowel *ME*. (n = 3 mice). Arrows indicate FOS-positive SOX10-EGCs. Scale bar (100 µm). **(B)** Confocal images of immunohistological stainings of SOX10 (magenta) and a rabbit IgG (green) in whole mounts of naïve small bowel *ME*. (n = 3 mice). Scale bar (100 µm). **(C)** Whole mounts of small bowel *ME* at naïve and Lap24h time points stained for myeloperoxidase^+^ cells. Scale bar (100 µm). **(D)** GIT (mean ± SEM) for naïve, Lap24h, and IM24h treated mice (n = 4-12 mice per condition; Student’s t-test, * to naïve, ### to Lap24h, ***/### < 0.001).


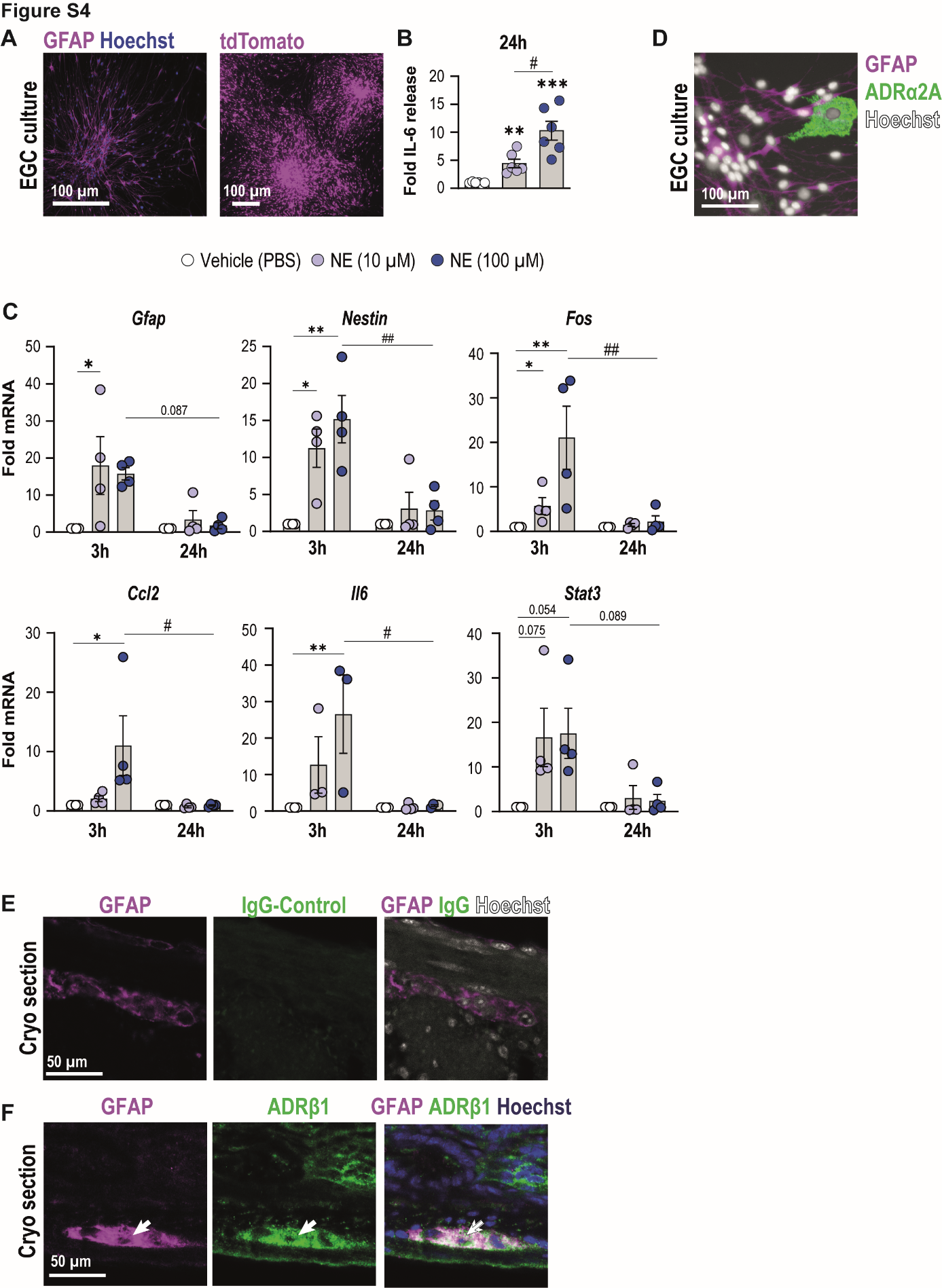


**Figure S4. Effect of NE and tissue expression of ADRβ1.**

**(A)** Immunocytochemistry images (Hoechst = blue; GFAP/tdTomato = magenta) of cultured primary EGCs. Scale bars (100 µm). **(B)** ELISA analysis for IL-6 release from conditioned medium (mean ± SEM) of cultured EGCs after NE stimulation (24 h; 10 µM, 100 µM; n = 6-8 distinct cell culture wells per condition; Student’s t-test, * to naive, # to 10 µM treatment, #/* < 0.05, ** < 0.01, *** < 0.001). **(C)** qPCR analysis of RNA from cultured EGCs (mean ± SEM) after NE (10 µM, 100 µM) treatment for 3h and 24 h for acute gliosis (2^‑ΔΔCT^, *18S*, untreated control; n = 4 distinct cell culture wells per condition; two-way ANOVA, * to the respective vehicle, # to 3h treatment, **/## < 0.01, */# < 0.05). **(D)** Immunocytochemistry of cultured EGCs for GFAP (magenta), Hoechst (white), and adrenergic receptor α2a (ADRα2A; green). Scale bar (100 µm). **(E)** Confocal images of immunohistochemistry of cryo-embedded intestinal samples stained for GFAP^+^enteric glia (magenta) and ADRβ1 (green) in the *ME*; Hoechst was used to detect cell nuclei (blue). Arrows indicate double positive cells. Scale bar (50 µm). **(F)** Confocal images of immunohistochemistry of cryo-embedded intestinal samples stained for GFAP^+^enteric glia (magenta) and IgG control (green) in the *ME*; Hoechst was used to detect cell nuclei (white). Scale bar (50 µm).

**Figure S5. *Ex vivo* β-adrenergic stimulation elicits enteric glial calcium signaling.**

**(A)** Grayscale snapshots from a 3D Ca^2+^ imaging sequence in which the glial cell network was identified by Substance P and the neurons by high K^+^ (upper panels) as well as fixed and immunohistochemically stained whole mounts for GCaMP3-GFP (green), glial cell network (GFAP, teal), and neuronal network (HuC/D, red) (lower panels). Large arrows indicate enteric glia reacting to Substance P, arrow heads indicate neurons reacting to high K^+^ levels. Scale bar (100 µm). **(B)** Average traces of 14 glial cells responding to Substance P (red) and isoprenaline (green). Note that these applications were done sequentially and then overlaid in time for presentation purposes. **(C)** Grayscale snapshot of a live recording of a maximal cytosolic response to SP, perfectly matching the glial network from a GFAP staining. Arrows indicate HuC/D positive cells that are not active at the time the glial cell network is during the peak SP response. Scale bar (100 µm). **(D)** Schematic of the administration of reserpine (100 µl s.c., 20 mg/kg body weight), subsequent NE depletion and laparotomy 24h after injection, and workflow. **(E)** qPCR analysis of RNA from *ME* (mean ± SEM) 3h after laparotomy of animals either untreated or treated with reserpine 24h before surgery for acute gliosis markers (2 ΔΔCT, 18S, NaCl 3h; n = 4 for Lap3h, n = 8 for Lap3h+Reserpine). **(F)** Schematic of the administration of tyramine (100 µl i.p., 100 mg/kg body weight), subsequent NE release and workflow. **(G)** qPCR analysis of RNA from *ME* (mean ± SEM) 3h after NaCl or tyramine treatment for acute gliosis markers (2 ΔΔCT, 18S, NaCl 3h; n = 4 for NaCl 3h, n = 8 for tyramine 3h).

**Figure S6. *JellyOP* mice with a mixed genetic background develop regular POI.**

**(A)** Confocal images of SOX10 (magenta) and JellyOP-GFP (green) expression in *Sox10^iCreERT2^/Rpl22^HA/+^/Ai14^fl/+^/JellyOP^fl/+^* mice. Arrows indicate GFP^+^/Sox10^+^ enteric glia. Scale bar (100 µm). **(B)** Confocal images of FOS (green) and SOX10 (magenta) in whole mounts of *Sox10^iCreERT2^/Rpl22^HA/+^/Ai14^fl/+^/JellyOP^fl/+^* mice with and without the *JellyOP* construct 3h after stimulation with blue light and under naïve conditions. Arrows indicate FOS^+^/SOX10^+^ enteric glia. Asterisks indicate oblong-shaped FOS^+^ smooth muscle cells. Diamonds indicate FOS^+^/SOX10^-^ ganglionic cells, likely enteric neurons. Scale bar (100 µm). **(C)** Confocal images of FOS (green) and SOX10 (magenta) in whole mounts of *Sox10^iCreERT2^/Rpl22^HA/+^/Ai14^fl/+^/JellyOP^fl/+^* mice 3h after intestinal manipulation. Arrows indicate FOS^+^/SOX10^+^ enteric glia. Scale bar (100 µm). **(D)** Whole mounts of small bowel *ME* of *Sox10^iCreERT2^/Rpl22^HA/+^/Ai14^fl/+^/JellyOP^fl/+^* mice at naïve and IM24h time points stained for myeloperoxidase^+^ cells. Scale bar (100 µm). **(E)** Quantification of myeloidperoxidase^+^ (MPO) cells/mm^2^ (mean ± SEM; n = 8-9 mice per condition; Student’s t-test, *** < 0.001). **(F)** Fluorescent images of SOX10 (magenta) and Ki67 (green) expression in whole mounts of small bowel *ME* in naïve and IM24h *Sox10^iCreERT2^/Rpl22^HA/+^/Ai14^fl/+^/JellyOP^fl/+^* mice. Scale bar (100 µm). **(G)** Histological analysis of Ki67^+^/SOX10^+^ cells per field of view (n = 4-5 animals per time point; mean counts of 5 images per n ± SEM; Student’s t-test, *** < 0.001). **(H)** GIT (mean ± SEM) for naïve and IM24h treated *Sox10^iCreERT2^/Rpl22^HA/+^/Ai14^fl/+^/JellyOP^fl/+^* mice (n = 5 mice per condition; Student’s t-test, *** < 0.001).
